# Fecal microbiota transplantation ameliorates alcohol-associated liver disease through coordinated restoration of short-chain fatty acid and *α*-linolenic acid signaling

**DOI:** 10.3389/fmicb.2026.1744446

**Published:** 2026-03-11

**Authors:** Rong Su, Junbai Ma, Jingyu Li, Yuanyuan Liu, Tian Ma, Jing Wang, Qian Mai, Qian Ma, Jingjing Wang, Hao Wang, Shaoqi Yang, Xiaoxia Zhang

**Affiliations:** 1 Department of Gastroenterology, First Clinical Medical College, General Hospital, Ningxia Medical University, Yinchuan, Ningxia, China; 2 School of Basic Medical Sciences, Ningxia Medical University, Yinchuan, Ningxia, China; 3 Department of Thoracic Surgery, General Hospital of Ningxia Medical University, Yinchuan, Ningxia, China; 4 .Research Department, General Hospital, Ningxia Medical University, Yinchuan, Ningxia, China; 5 Medical Laboratory Department, General Hospital, Ningxia Medical University, Yinchuan, Ningxia, China; 6 College of Traditional Chinese Medicine, Ningxia Medical University, Yinchuan, Ningxia, China

**Keywords:** alcohol-associated liver disease, fecal microbiota transplantation, GPR43, gut–liver axis, metabolomics, PPAR*α*, short-chain fatty acids, *α*-linolenic acid

## Abstract

**Background:**

Alcohol-associated liver disease (ALD) is closely linked to gut microbiota dysbiosis. However, the specific microbial metabolic functions that drive the transition from microbial imbalance to hepatic inflammation and metabolic injury remain unclear, limiting the development of mechanism-based therapeutic strategies.

**Methods:**

This study integrated human microbiome analysis with fecal microbiota transplantation (FMT) experiments in an ALD mouse model. Multi-omics approaches, including 16S rRNA gene sequencing, untargeted metabolomics, and immunological profiling, were employed to systematically characterize the interactions among gut microbiota composition, microbial-derived metabolites, and host immune responses.

**Results:**

We observed that ALD progression was characterized by an early shift in microbial composition followed by a marked decline in microbial diversity, culminating in an ecological collapse of the gut microbiota. FMT from healthy donors significantly improved liver histopathology and serum biochemical parameters, accompanied by restoration of gut microbial diversity and key metabolic functions. Metabolomic analyses revealed enhanced short-chain fatty acid (SCFA) production and activation of *α*-linolenic acid (ALA)-related metabolic pathways following FMT. These metabolic improvements were associated with reduced inflammatory responses and improved immune homeostasis.

**Conclusion:**

Our findings demonstrate that FMT from healthy donors ameliorates ALD by restoring critical microbial metabolic functions, particularly SCFA production and ALA-related pathways. These results highlight microbial metabolic function as a promising therapeutic target for microbiome-based interventions in ALD.

## 1 Introduction

Alcohol-associated liver disease (ALD) poses a formidable global health burden, with its progression from steatosis to hepatitis and cirrhosis driven in large part by a dysfunctional gut–liver axis (Szabo and Bala, 2010; Hsu and Schnabl, 2023). While gut dysbiosis and impaired barrier function are established hallmarks of ALD, the specific microbial metabolites that translate these defects into hepatic inflammation remain incompletely mapped.

Fecal microbiota transplantation (FMT) has shown promise in early clinical reports and systematic reviews for severe alcoholic hepatitis (AH), providing preliminary human evidence for microbiome-targeted interventions (Shasthry, 2020; Philips et al., 2017). However, the precise mechanisms bridging microbial engraftment to host immunometabolic improvement remain largely unexplored, and unlocking this mechanistic “black box” is critical for transforming FMT from an empirical therapy into a targeted, mechanism-based intervention.

We hypothesized that the therapeutic effect of FMT is mediated through the restoration of key microbial metabolites that directly regulate host inflammatory and metabolic pathways. As previously mentioned, experimental and early clinical evidence has demonstrated that FMT can prevent or ameliorate alcohol-related liver injury. However, the core of this study lies in uncovering new mechanistic insights behind the known fact that FMT improves phenotypic manifestations. Based on this, we focus on two key classes of microbiota-derived metabolites: short-chain fatty acids (SCFAs), such as butyrate (Liu et al., 2021) and *α*-linolenic acid (ALA), an *ω*-3 polyunsaturated fatty acid (Liu et al., 2023). Existing research has revealed that SCFAs can modulate the structure of the gut microbiota and enhance the function of the intestinal epithelial barrier (Zhang et al., 2023). Furthermore, SCFAs exert anti-inflammatory effects and maintain intestinal barrier function by activating G-protein-coupled receptor 43 and inhibiting histone deacetylases (Tao and Wang, 2025; Li et al., 2025; Huang et al., 2024). Other *ω*-3 fatty acids, such as ALA, can exert anti-inflammatory effects in Kupffer cells by activating their receptors (GPR120/FFA4), thereby improving alcoholic hepatic steatosis (Kang et al., 2024).

We propose the scientific hypothesis that the ameliorative effect of FMT on ALD may depend on its ability to coordinately restore the biosynthesis and balance of SCFAs and ALA in the gut, thereby synergistically improving the disease process at multiple levels. This study aims to systematically validate this hypothesis by integrating multi-omics approaches, including metagenomics, metabolomics, and molecular biology, to elucidate the novel mechanisms underlying FMT treatment for ALD and provide new evidence for the development of precision therapeutic strategies based on microbial metabolite signaling.

## 2 Materials and methods

### 2.1 Ethics approval

Fecal samples used for gut microbiota analysis were collected from newly diagnosed patients with AH and alcohol-associated cirrhosis (AC) between October 31, 2023 and May 1, 2024. In addition, 39 age- and sex-matched healthy volunteers were enrolled as controls. The distribution of samples across groups was unequal (AH, *n* = 8; AC, *n* = 23; Healthy, *n* = 39), reflecting real-world clinical recruitment conditions (Supplementary Table S1). Compared with AC patients and healthy individuals, AH patients were in an acute and often severe clinical state, which imposed stricter ethical constraints on invasive sampling. Moreover, their shorter hospitalization duration and clinical instability limited the availability of qualified fecal specimens. As the primary objective of the human cohort analysis was to characterize the ecological background of donor-derived microbiota and inform FMT donor stratification and functional investigation, rather than to perform population-level statistical inference, no forced sample size balancing was applied. All comparative analyses were conducted using non-parametric statistical methods, and interpretations were made cautiously in consideration of group size differences.

The study protocol was approved by the Ethics Committee of the General Hospital of Ningxia Medical University (Approval No. KYLL-2024-1064), and all participants provided written informed consent. Disease diagnosis and clinical staging were performed in accordance with the 2018 guidelines issued by the Chinese Medical Association (Li et al., 2025; Huang et al., 2024).

Inclusion criteria: (i) age ≥ 18 years; (ii) a confirmed history of alcohol consumption, with documented duration, frequency, and quantity of intake; (iii) complete clinical and demographic data; and (iv) agreement to longitudinal follow-up. Exclusion criteria: (i) incomplete documentation of alcohol use history; (ii) severe non-alcohol-related comorbidities affecting survival outcomes; (iii) pregnancy or lactation; (iv) inadequate follow-up duration; and (v) use of medications known to markedly alter gut microbiota composition within 3 months prior to sample collection, including antibiotics, glucocorticoids, N-acetylcysteine, or heparin.

**Figure 1:**
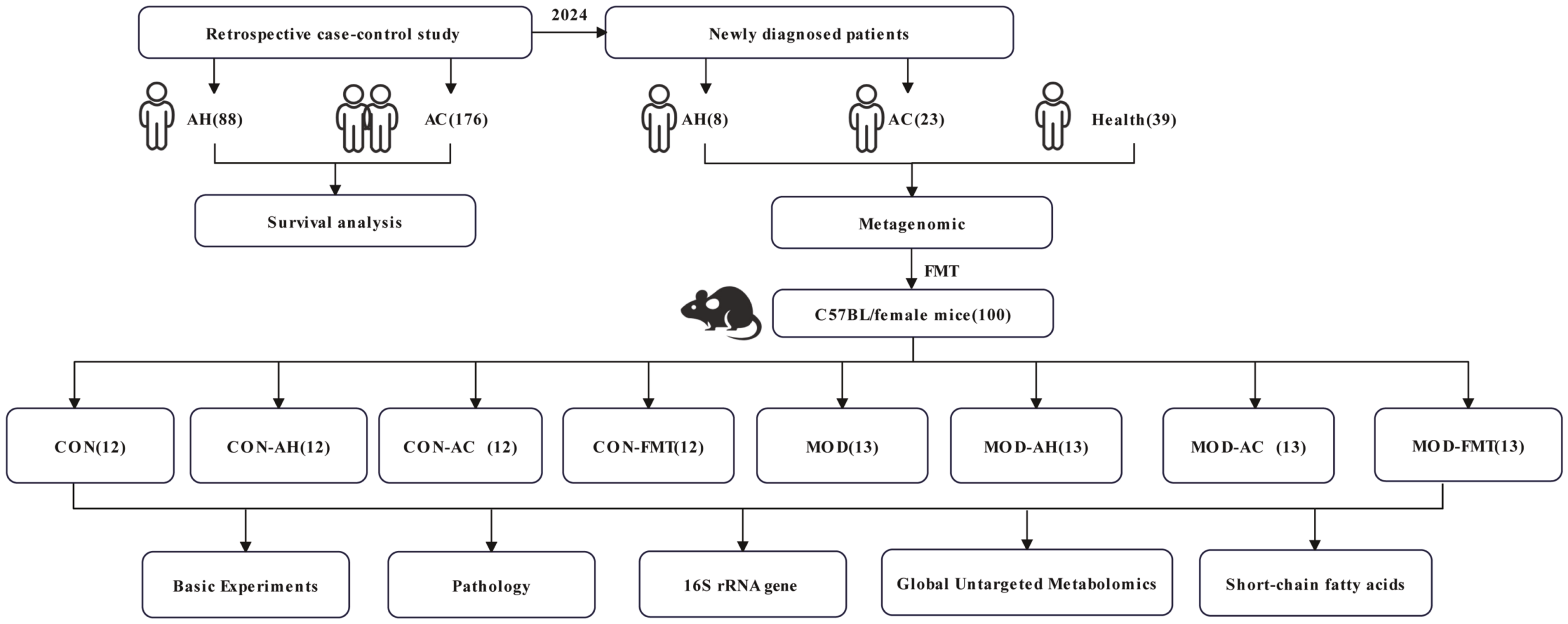
Overview of the experimental and analytical workflow.

All participants underwent sample collection after a 12 h overnight fast. Rectal swabs and fecal samples were collected using sterile cryogenic tubes, and immediately stored at −80 °C until further analysis. Figure 1 illustrates the overall technical workflow of this study.

### 2.2 Mouse model and FMT interventions

All animal experiments were approved by the Experimental Animal Ethics and Welfare Committee of the General Hospital of Ningxia Medical University (IACUA-NYLAC-2024-069) and conducted in accordance with ARRIVE 2.0 guidelines. Female C57BL/6 J mice (6 weeks old) were housed under SPF conditions (22 ± 2 °C, 12 h light/dark cycle) and fed either a modified Lieber-DeCarli ethanol diet or an isocaloric control diet.

Sample size and randomization: *A priori* power analysis (ɑ = 0.05, power = 0.80, eight groups) indicated that at least 12 mice per group were required to detect a medium effect size for the primary biochemical outcomes. To account for potential attrition, 13 mice per group were included. Mice were randomly assigned to eight groups using a computer-generated randomization sequence. Histological, imaging, ELISA, and flow cytometry assessments were performed by investigators who blinded to group allocation until completion of the analyses.

Antibiotic pretreatment and grouping: All mice received broad-spectrum antibiotic pretreatment to deplete endogenous gut microbiota. Antibiotics were dissolved and combined to final concentrations of 5 mg/mL ampicillin, 5 mg/mL neomycin sulfate, 5 mg/mL metronidazole, and 2.5 mg/mL vancomycin. The mixture was administered by oral gavage at 0.5 mL/100 g body weight once daily for 7 consecutive days. After antibiotic withdrawal, mice were maintained on a standard diet for 1 week prior to further interventions. Subsequently, C57BL/6 J mice were randomly assigned to eight groups (*n* = 13 per group) as follows: Negative control group (CON): fed a modified Lieber–DeCarli liquid diet without ethanol. CON/AH group: fed a modified Lieber–DeCarli liquid diet without ethanol and received FMT (200 μL) derived from patients with AH. CON/AC group: fed a modified Lieber–DeCarli liquid diet without ethanol and received FMT (200 μL) derived from patients with AC. CON/FMT group: fed a modified Lieber–DeCarli liquid diet without ethanol and received FMT (200 μL) from qualified healthy standardized donors. Model group (MOD): fed a modified Lieber–DeCarli ethanol-containing liquid diet to establish ALD. MOD/AH group: fed the ethanol-containing modified Lieber–DeCarli diet and received AH-derived FMT (200 μL). MOD/AC group: fed the ethanol-containing modified Lieber–DeCarli diet and received AC-derived FMT (200 μL). MOD/FMT group: fed the ethanol-containing modified Lieber–DeCarli diet and received FMT (200 μL) from qualified healthy standardized donors. FMT was administered by oral gavage for two consecutive weeks and then discontinued. Mice subsequently continued on their respective ethanol-containing or isocaloric control diets. The ALD model was established using a modified Lieber–DeCarli liquid diet. In the ethanol group, the dietary energy composition consisted of 18% protein, 19% carbohydrate, 35% fat, and 28% ethanol. The control group received an isocaloric liquid diet in which ethanol-derived calories were replaced with an equivalent caloric amount of maltodextrin to ensure equal total energy intake across groups. After 6 weeks of feeding, mice were euthanized, and relevant endpoints were assessed.

Preparation of bacterial suspensions: Fresh stool samples from AH and AC patients or healthy donors were processed within 2 h of collection. Donors were matched as closely as possible with respect to disease classification, age range, and clinical characteristics to minimize confounding variability. Approximately 2 g of stool was homogenized in 20 mL sterile saline, filtered through 200- and 300-mesh strainers, centrifuged sequentially at 400 × g for 5 min (three times) and then at 6,000 × g for 5 min. The resulting pellet was resuspended in 10 mL sterile saline (1 g stool equivalent per 15% glycerol-LB medium). Prior to use, all donor material underwent standard clinical screening, including medical history questionnaire assessment, serological testing, stool pathogen screening, and 16S rRNA-based microbial diversity analysis. To reduce inter-individual variability and improve reproducibility, stool samples from three donors per group were pooled before preparation, consistent with previously published FMT animal studies. Preparation procedures, concentration adjustment, storage conditions, and gavage volumes were standardized across experimental batches. Prepared bacterial suspensions were stored at −80 °C to ensure traceability and batch consistency.

FMT administration: Mice received 200 μL bacterial suspension (from AH, AC, or healthy donors) by intragastric gavage once daily for 2 consecutive weeks, followed by continued diet administration for 6 weeks. Fluid intake, body weight, and behavioral status were monitored daily, and humane endpoints were predefined. After completion of FMT treatment, relevant outcome measures were assessed.

### 2.3 Biological and biochemical analyses

Histological staining (Hematoxylin and eosin (H&E) and Oil Red O): Immediately after harvesting, liver tissues were fixed in 4% paraformaldehyde for 24 h. Samples were subsequently dehydrated through graded ethanol, cleared in xylene, and embedded in paraffin. Paraffin blocks were sectioned at a thickness of 4 μm. H&E staining was performed to evaluate hepatic architecture and inflammatory cell infiltration, following established protocols described previously. For lipid accumulation assessment, Oil Red O staining was conducted on frozen liver sections (8 μm). Cryosections were briefly fixed in 10% neutral formalin prior to incubation with Oil Red O working solution (Sigma-Aldrich, Cat# O0625) for 15 min. Nuclei were counterstained with hematoxylin. All stained sections were imaged using a Leica DM6 B optical microscope (Leica Microsystems). Image acquisition and quantitative evaluation were conducted by investigators blinded to group allocation.

Intestinal immunofluorescence staining: Colon tissues were fixed in 4% paraformaldehyde, embedded in paraffin, and sectioned at 4 μm thickness. After deparaffinization and rehydration, antigen retrieval was performed using citrate buffer (pH 6.0) with microwave heating. Following blocking, sections were incubated with primary antibodies against Occludin (Thermo Fisher Scientific, Cat# 71-1500) and Claudin-4 (Abcam, Cat# ab53156), followed by appropriate fluorescence-conjugated secondary antibodies. Nuclear counterstaining was achieved with DAPI. Fluorescent images were captured using a Leica SP8 confocal microscope and quantified using ImageJ software to determine relative fluorescence intensity.

Serum biochemical measurements: Blood samples were collected via orbital venous plexus puncture and centrifuged at 4 °C (3,000 rpm, 10 min) to obtain serum. Serum levels of aspartate aminotransferase (AST), alanine aminotransferase (ALT), and alkaline phosphatase (AKP) were measured using an automated biochemical analyzer (Hitachi 7600-120, Hitachi High-Technologies, Japan) according to the manufacturer’s instructions. Total bilirubin (TBIL), direct bilirubin (DBIL), total bile acids (TBA), and triglycerides (TG) were quantified using enzyme-linked immunosorbent assay (ELISA) kits.

ELISA and lipopolysaccharide (LPS) quantification: Concentrations of TNF-*α*, IFN-*γ*, IL-6, IL-17A, and IL-10 in plasma or tissue homogenates were determined using commercially available ELISA kits, including TNF-α (BioLegend, Cat# 430901), IFN-γ (BioLegend, Cat# 430801), IL-6 (BioLegend, Cat# 431301), IL-17A (BioLegend, Cat# 432501), and IL-10 (BioLegend, Cat# 431401). Liver tissues were homogenized in RIPA lysis buffer, and cytokine levels were normalized to total protein concentrations. Hepatic LPS levels were measured using a limulus amebocyte lysate (LAL) assay kit (Lonza, Cat# QCL-1000) in strict accordance with the manufacturer’s protocol.

Flow cytometry: All flow cytometric analyses were conducted using a standardized gating and quality control strategy. Initially, the cell population was identified based on forward and side scatter (FSC/SSC) parameters to exclude debris. Dead cells were removed using the Zombie Aqua Fixable Viability Kit (BioLegend, Cat# 423101). Doublets and aggregates were excluded by singlet discrimination using FSC-A versus FSC-H (or FSC-W) gating. Fluorescence compensation was established for each experiment using single-stained compensation beads (BD CompBeads, BD Biosciences) or single-stained cell controls from the same batch to generate the compensation matrix. Fluorescence-minus-one controls were included in every run to accurately define gating thresholds for key markers, including iNOS, CD206, Foxp3, IFN-*γ*, and IL-17A. Data acquisition was performed on a BD LSRFortessa X-20 flow cytometer (BD Biosciences). At least 1 × 10^5 live singlet events were collected per sample. Data were analyzed using FlowJo software (v10.8.1, BD Biosciences). Analysis of hepatic macrophage polarization: Within the live singlet population, macrophages were defined as CD11b^+F4/80^+ cells (CD11b-APC, clone M1/70, BioLegend, Cat# 101212; F4/80-PE, clone BM8, BioLegend, Cat# 123110). M1 and M2 polarization markers were subsequently assessed within this gated population. M1 macrophages were identified by intracellular iNOS expression (iNOS-APC, clone CXNFT, Invitrogen/eBioscience, Cat# 17-5920-82), whereas M2 macrophages were characterized by surface CD206 expression (CD206-PB450, BioLegend, Cat# 141717). Polarization ratios were quantified using consistent quadrant or threshold gating strategies across experiments. Splenic T Cell, Treg, and Th17 phenotyping: Within live singlets, CD3^+CD4^+ T cells were sequentially gated (CD3ε-PerCP-Cy5.5, clone 145-2C11, BioLegend, Cat# 100328; CD4-FITC, clone GK1.5, BioLegend, Cat# 100406). Regulatory T cells (Tregs) were strictly defined within the CD4^+ compartment as CD25^+Foxp3^+ cells (CD25-APC, BioLegend, Cat# 102012). Intracellular Foxp3 staining was performed following fixation and permeabilization using the Foxp3/Transcription Factor Staining Buffer Set (eBioscience, Cat# 00-5523-00), with Foxp3-PE antibody (clone FJK-16 s, eBioscience, Cat# 12-5773-82). Intracellular Cytokine Staining (ICS) for IFN-*γ* and IL-17A: To avoid false-negative results due to minimal cytokine production under unstimulated conditions, intracellular cytokine staining was performed following ex vivo stimulation. Single-cell suspensions were incubated in complete culture medium containing PMA (50 ng/mL, Sigma-Aldrich, Cat# P8139) and ionomycin (1 μg/mL, Sigma-Aldrich, Cat# I0634) for 4 h. Brefeldin A (5 μg/mL, BioLegend, Cat# 420601) was added at the initiation of stimulation to block cytokine secretion. Unstimulated controls were processed in parallel and also treated with Brefeldin A to control for background signal. After stimulation, surface staining for CD3 and CD4 was performed, followed by fixation and permeabilization using the same Foxp3 staining buffer set described above. IFN-*γ* and IL-17A expression were quantified within the CD3^+CD4^+ T cell population (IFN-γ-APC, clone XMG1.2, BioLegend, Cat# 505810; IL-17A-APC-Cy5.5, used according to the predefined antibody panel and instrument configuration, with clone and catalog numbers detailed in the Supplementary Table “Antibody panel for flow cytometry”).

### 2.4 Microbiome and metabolomics

Metagenomics: DNA was extracted from fecal samples using the PF Mag-Bind kit. Sequencing libraries were prepared using the NEXTFLEX system and sequenced on an Illumina NovaSeq platform. Reads were quality-filtered (fastp), assembled (MEGAHIT), and open reading frames predicted with Prodigal.

16S rRNA amplicon sequencing: V3-V4 regions (338F/806R) were sequenced (PE300 mode), denoised using DADA2 (QIIME 2), and taxonomically classified against the SILVA v138 database. Functional prediction was performed using PICRUSt2.

SCFAs: Approximately 50 mg of fecal material was acidified with phosphoric acid, and SCFAs were extracted using anhydrous ether, with 4-methylpentanoic acid serving as the internal standard. Samples were analyzed using a gas chromatography–mass spectrometry system (Agilent 7890B GC coupled with 5977A MSD). Separation was performed on an HP-FFAP capillary column (30 m × 0.25 mm × 0.25 μm). High-purity helium was used as the carrier gas at a flow rate of 1.0 mL/min. The injector temperature was set at 250 °C. The oven temperature was initially maintained at 80 °C for 1 min, then increased to 180 °C at a rate of 10 °C/min and held for 5 min. Mass spectrometric detection was conducted under electron impact ionization at 70 eV, with a scanning range of m/z 50–500. A pooled quality control (QC) sample was analyzed after every ten samples to monitor instrument stability, and QC samples with relative standard deviation (RSD) values below 15% were considered acceptable. SCFA concentrations were normalized to the wet weight of fecal or tissue samples. All raw GC–MS data have been deposited in the NCBI database under accession number PRJNA1308792.

Untargeted metabolomics: LC–MS/MS analysis was conducted using UHPLC-Orbitrap in both positive and negative ionization modes. Pooled QC samples were analyzed at regular intervals. RSD values <15% were accepted.

Data deposition: Raw metagenomic reads have been deposited in NCBI under accession number PRJNA1308792. Processed datasets and analysis scripts will be available in a public repository upon article acceptance.

### 2.5 Statistical analysis

Analyses were performed using R (version ×4.5) and GraphPad Prism (version ×9.0). Continuous variables are expressed as mean ± SD or median (IQR) as appropriate. Normality was assessed by the Shapiro–Wilk test and homogeneity of variance by Levene’s test. Two-group comparisons were performed with Student’s *t*-test (parametric) or Mann–Whitney U test (non-parametric). Multiple-group comparisons were analyzed with one- or two-way ANOVA with Tukey’s post-hoc test (parametric) or Kruskal–Wallis with Dunn’s correction (non-parametric). Microbiome analyses: Comparisons of *α*-diversity among more than two groups were performed using the Kruskal–Wallis test followed by Dunn’s *post hoc* test. Differences in *β*-diversity were assessed by PERMANOVA based on Bray–Curtis distance with 999 permutations. Multiple comparisons were adjusted using the Benjamini–Hochberg method to control the false discovery rate (FDR). Differential taxa were identified by linear discriminant analysis effect size (LEfSe) (LDA > 3.5) with Benjamini–Hochberg FDR control and corroborated using ANCOM-BC or DESeq2. Spearman’s rank correlation coefficient (*ρ*) was used to assess pairwise correlations with FDR adjustment for multiple testing. Survival analysis was performed using Kaplan–Meier curves with log-rank testing; variables with *p* < 0.10 in univariate analysis were entered into multivariable Cox regression. Two-sided *p* < 0.05 (or FDR q < 0.10 for multi-omics analyses) was considered statistically significant. Effect sizes and exact sample sizes (*n*) are reported in the corresponding figure legends and tables.

## 3 Results

### 3.1 Stage-specific gut dysbiosis in ALD

**Figure 2:**
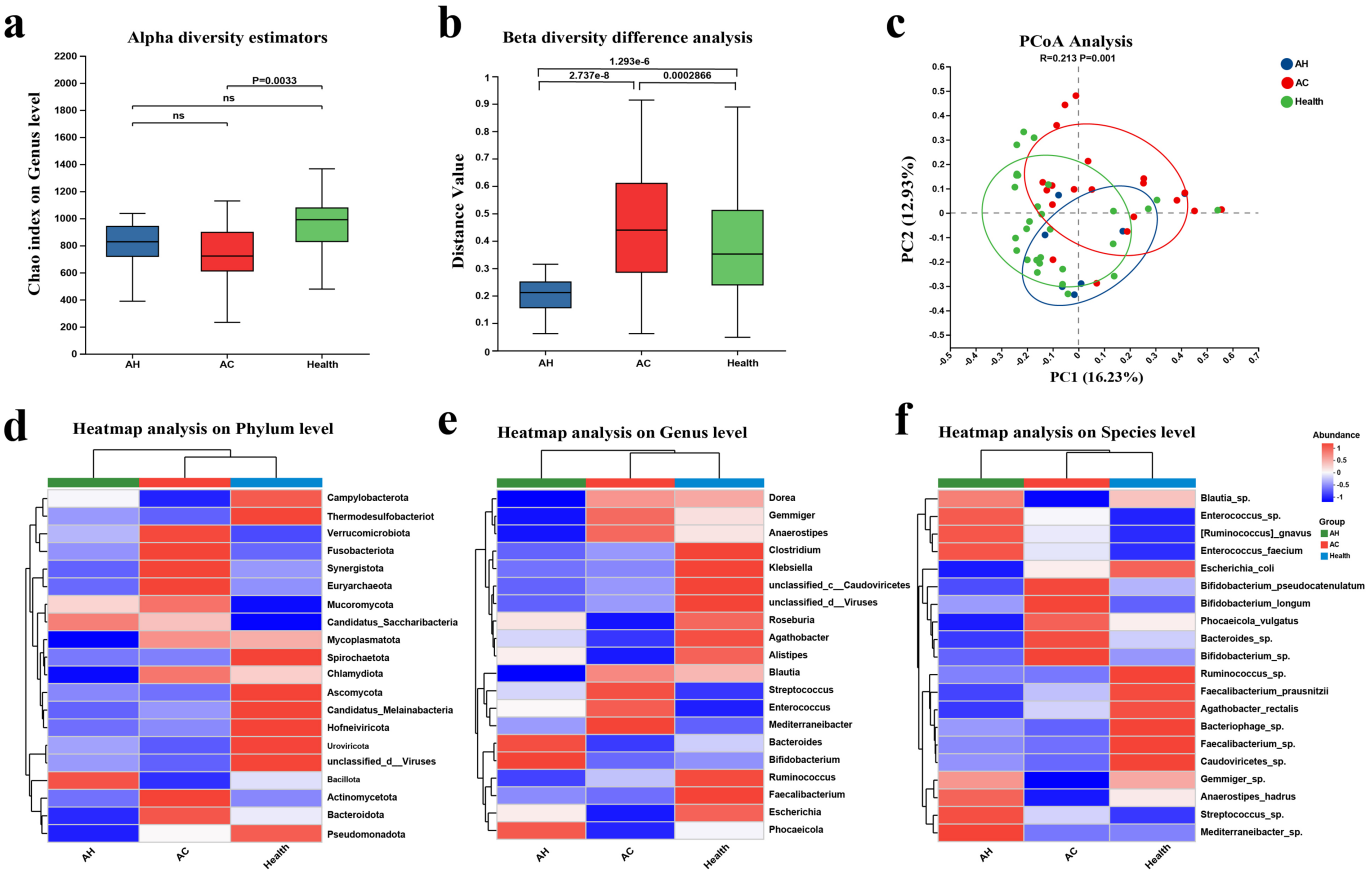
Gut microbiota composition analysis. **(a)** Alpha diversity analysis: species richness of the gut microbiota in healthy controls (health), AH, and AC was evaluated using the Chao1 index. Intergroup comparisons were performed using the Kruskal–Wallis test followed by Dunn’s *post hoc* test, with multiple comparisons corrected by FDR adjustment. **(b)** Beta diversity analysis: Community dissimilarity among groups was calculated based on Bray–Curtis distances. Statistical significance of intergroup differences was assessed using permutational multivariate analysis of variance (PERMANOVA). **(c)** Principal coordinates analysis (PCoA): PCoA based on Bray–Curtis distances was conducted to visualize differences in community structure among groups. The analysis revealed partial separation among groups with some degree of overlap. **(d–f)** Taxonomic composition heatmaps at multiple hierarchical levels: Relative abundance profiles of gut microbiota were displayed at the phylum **(d)**, genus **(e)**, and species **(f)** levels to characterize stage-specific remodeling of community structure across the three groups. Colors represent standardized relative abundance values.

To characterize microbial alterations across disease stages, we compared the gut microbiota of patients with AH, AC, and healthy controls (Figure 2).

#### 3.1.1 Microbial diversity

Alpha diversity declined progressively from AH to AC. Compared with healthy controls, the AC group exhibited a significant reduction in *α*-diversity (Wilcoxon AC vs. Health, *p* = 0.0033), indicating that disease progression is accompanied by impaired overall microbial richness. In contrast, α-diversity in the AH group remained comparable to that of healthy controls; however, substantial alterations in community structure were already evident. Beta diversity analysis further demonstrated stage-associated community restructuring. Significant differences in microbial composition were observed between AH and AC, AH and healthy controls, as well as AC and healthy controls (AH vs. AC, *p* = 2.7 × 10^−8^; AH vs. Health, *p* = 1.3 × 10^−6^; AC vs. Health, *p* = 0.0003). Principal-coordinates analysis confirmed distinguishable clustering patterns among groups in low-dimensional space, although partial overlap was present, indicating that *β*-diversity shifts reflect population-level structural displacement rather than complete segregation (PERMANOVA overall *p* = 0.001; Figures 2a–c).

#### 3.1.2 Taxonomic shifts

Heatmaps depicting overall taxonomic abundance at the phylum, genus, and species levels demonstrated systematic remodeling of the gut microbiota in both AH and AC stages. Distinct compositional patterns were observed across disease stages, reflecting progressive ecological restructuring (Figures 2d–f).

#### 3.1.3 Stage-specific signatures

LEfSe analysis was performed to systematically identify differentially abundant taxa. The resulting cladogram illustrated taxa significantly enriched in the AH, AC, and healthy control groups, along with their phylogenetic relationships (Supplementary Figure S2a), while LDA were used to quantify the contribution of each discriminative taxon to group separation (Supplementary Figure S2b). In addition, representative differential taxa at the phylum, genus, and species levels were selected to compare their relative abundances across groups. Several taxa exhibited stage-related trends among healthy controls, AH, and AC (Supplementary Figures S2c,d). These analyses were primarily intended to provide a descriptive overview of stage-associated taxonomic differences rather than to support mechanistic conclusions.

Collectively, these data indicate that AH is characterized primarily by compositional reorganization of the microbiota, whereas AC reflects a more profound ecological collapse marked by pathogen enrichment and depletion of beneficial taxa.

### 3.2 Functional consequences of ALD-associated gut dysbiosis

**Figure 3:**
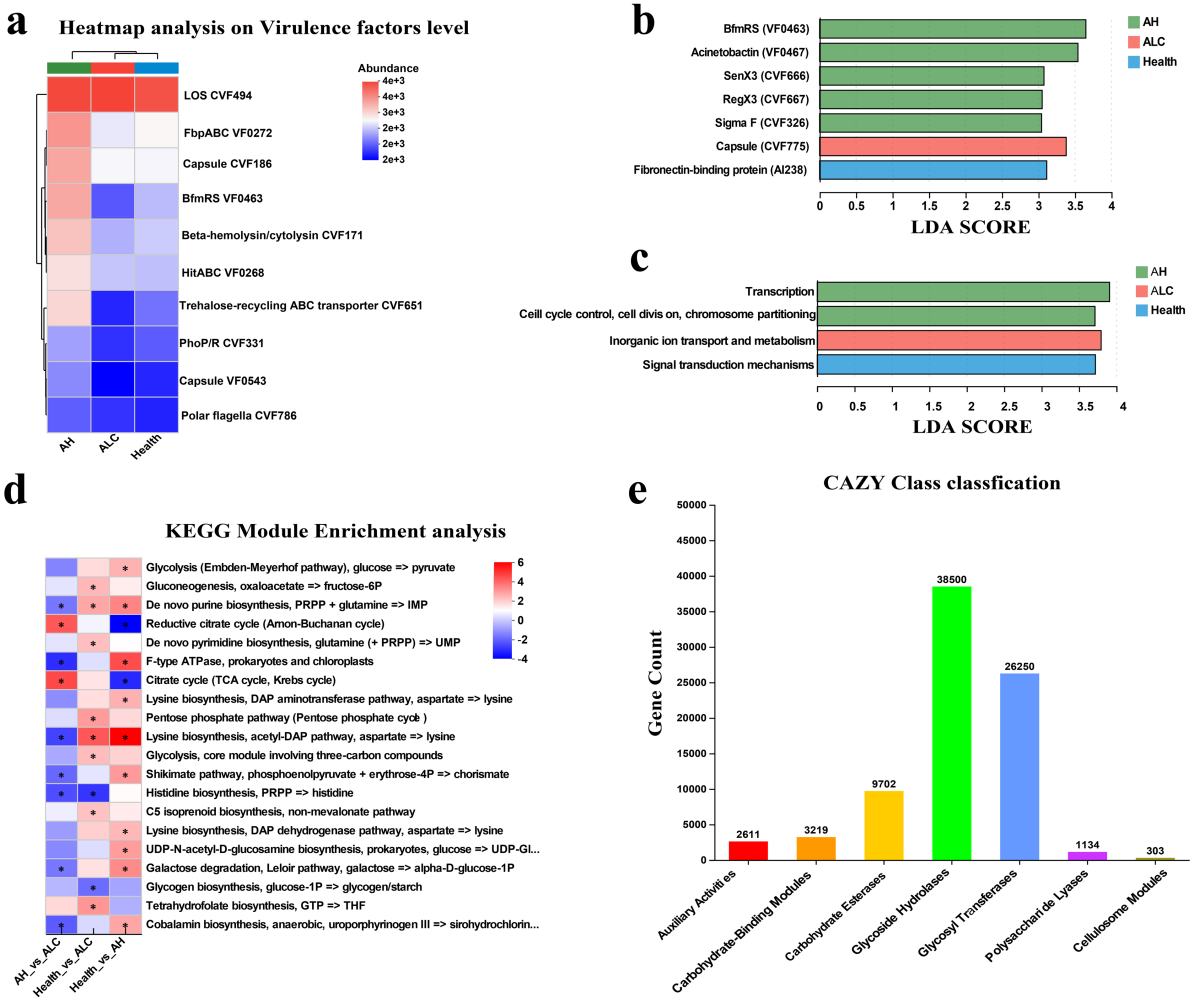
Functional characterization of microbial communities. **(a)** Heatmap of VF abundance. A heatmap illustrating the relative abundance of VFs across groups. **(b)** LEfSe analysis of VFDB annotations (LDA > 3). Differentially enriched VFs identified based on the VFDB using LEfSe analysis with an LDA score threshold > 3. **(c)** LEfSe analysis of clusters of orthologous groups (COG) functional categories (LDA > 2). Significantly discriminative COG functions identified among groups using LEfSe with an LDA score threshold > 2. **(d)** Differential microbial gene functions between AH, ALC, and healthy control groups. Functional pathways enriched in healthy controls or post-treatment patients are shown in purple, whereas those enriched in pre-treatment patients are shown in red. Asterisks indicate pathways with reported gene scores > 1.65 or < −1.65. **(e)** Carbohydrate-active enzyme (CAZy) family classification: Distribution of CAZy families across groups, illustrating differences in microbial carbohydrate metabolism potential.

To further evaluate the functional alterations accompanying gut microbial dysbiosis across different stages of ALD, we systematically analyzed bacterial virulence factor (VF) profiles and multi-level metabolic functional characteristics (Figure 3). The heatmap of VF abundance demonstrated marked changes in the relative abundance of multiple virulence-related genes in AH and AC samples compared with healthy controls, suggesting a global remodeling of microbial pathogenic potential under disease conditions (Figure 3a). LEfSe analysis based on the Virulence Factor Database (VFDB) database (LDA > 3) further identified stage-specific enrichment patterns. In the AH group, regulatory or stress-associated factors such as BfmRS (VF0463), Acinetobactin (VF0467), SenX3 (CVF666), RegX3 (CVF667), and SigmaF (CVF326) were predominantly enriched, whereas in the AC group, capsule-associated proteins (CVF775) related to bacterial surface structures showed a higher enrichment trend (Figure 3b). At the functional metabolism level, LEfSe analysis of clusters of orthologous groups (COG) annotations (LDA > 2) revealed stage-dependent differences in core functional modules, including transcriptional regulation, cell cycle and chromosome segregation, inorganic ion transport, and signal transduction (Figure 3c).

KEGG module enrichment analysis further indicated that, compared with healthy controls, multiple pathways related to energy metabolism, amino acid biosynthesis, nucleotide metabolism, and molecular transport were significantly altered in ALD samples (FDR < 0.05), reflecting a broad restructuring of metabolic potential (Figure 3d). Additionally, carbohydrate-active enzyme (CAZy) functional profiling demonstrated substantial differences in enzyme categories such as glycoside hydrolases, glycosyltransferases, and carbohydrate-binding modules across disease stages, suggesting that the capacity of gut microbes to utilize complex carbohydrates changes during disease progression (Figure 3e; Supplementary Figure S3a). Integration with weighted gene co-expression network analysis (WGCNA) identified several functional modules significantly associated with AH, AC, or healthy states. These modules exhibited distinct correlation patterns in pathways related to metabolism, virulence, and environmental adaptation (Supplementary Figure S3b). Collectively, these functional genomic analyses indicate that gut microbiota in different stages of ALD undergo not only compositional shifts but also systematic remodeling of virulence profiles and metabolic capacity, thereby providing a functional framework for subsequent investigation of specific metabolite alterations and their association with host pathological status.

### 3.3 Therapeutic impact of FMT in an ALD mouse model

To test this hypothesis and to separate microbiota effects from other human confounders, we established an ethanol-fed mouse model of ALD and performed FMT using microbiota from (i) screened healthy donors, (ii) AH patients, and (iii) AC patients. The following sections describe how healthy-donor FMT influenced disease outcomes in comparison with FMT from AH or AC patients: (3.1) liver pathology and perfusion, (3.2) serum biochemical markers, (3.3) systemic and local inflammation, and (3.4) intestinal barrier integrity.

#### 3.3.1 Healthy-donor FMT mitigates hepatic steatosis and improves liver perfusion

**Figure 4:**
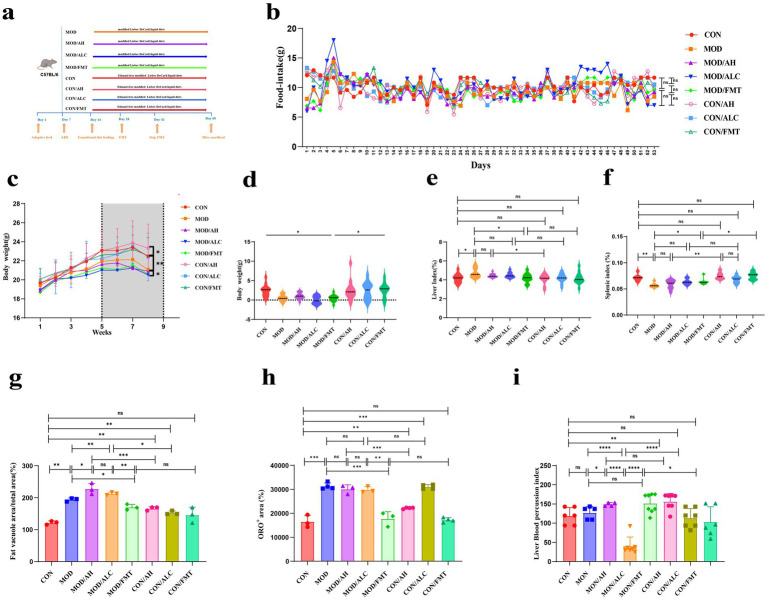
Phenotypic effects of FMT from different donor sources in an ALD mouse model. **(a)** Schematic illustration of the experimental design. Female C57BL/6 J mice received antibiotic pretreatment followed by ethanol or control diet feeding and were subsequently transplanted with fecal microbiota derived from healthy donors, AH patients, or AC patients. **(b)** Body weight changes under different dietary and FMT intervention conditions. **(c–f)** Organ index analysis. Liver and spleen indices were calculated to evaluate organ injury induced by ethanol feeding and FMT from different donor sources. **(g)** Steatosis scoring. **(h)** Quantification of hepatic lipid accumulation. **(i)** Quantitative analysis of hepatic perfusion assessed by laser speckle imaging. Data are presented as mean ± standard error of the mean (SEM). Intergroup comparisons were performed using one-way or two-way ANOVA, followed by appropriate post hoc multiple comparison tests when necessary. * *p* < 0.05, ** *p* < 0.01, *** *p* < 0.001.

To evaluate the biological effects of FMT from different donor sources in an ALD model, 100 female C57BL/6 J mice were randomly allocated into eight diet/FMT intervention groups. Following antibiotic-mediated microbiota depletion, mice received FMT derived from healthy donors, patients with AH, or patients with AC (three donors per group, pooled prior to preparation) (Figure 4a). Regarding general physiological parameters, ethanol-fed model (MOD) mice exhibited significantly reduced body weight compared with the CON group (*p* < 0.05). However, FMT from different donor sources did not significantly alter the overall body weight trajectory (Figure 4b). Organ index analysis demonstrated that healthy-donor FMT significantly reduced both liver index (*p* = 0.0205) and spleen index (*p* = 0.0407) in MOD mice, whereas FMT derived from AH or AC patients did not produce comparable improvements (Figures 4c–f). Histological assessment of hepatic lipid accumulation using H&E and Oil Red O staining revealed marked vacuolar degeneration and lipid droplet deposition in the MOD group. Healthy-donor FMT markedly alleviated these histopathological changes, whereas AH-FMT and AC-FMT were associated with aggravated steatosis (Supplementary Figures S4a,b). Quantitative analysis confirmed that healthy-donor FMT significantly reduced hepatic lipid accumulation (*p* < 0.05), while AH- and AC-derived FMT groups exhibited significantly elevated lipid deposition indices (*p* < 0.01) (Figures 4g,h). At the level of hepatic microcirculation, representative laser speckle imaging demonstrated donor-dependent differences in liver perfusion patterns (Supplementary Figure S4c). Quantitative analysis showed a trend toward improved hepatic perfusion following healthy-donor FMT that did not reach statistical significance (*p* = 0.0754). In contrast, AH/FMT increased perfusion in both MOD/AH (*p* = 0.0388) and CON/AH (*p* = 0.0097) conditions, whereas AC-FMT significantly reduced hepatic perfusion in the MOD/AC model (*p* < 0.0001) (Figure 4i). Collectively, these findings indicate that the phenotypic effects of FMT in the ALD model are strongly donor-dependent. Microbiota derived from healthy donors partially ameliorated ethanol-induced hepatic steatosis and organ injury, whereas patient-derived microbiota did not confer protective effects under the present experimental conditions and may even exacerbate liver pathology.

#### 3.3.2 Healthy-donor FMT improves serum biochemistry

**Figure 5:**
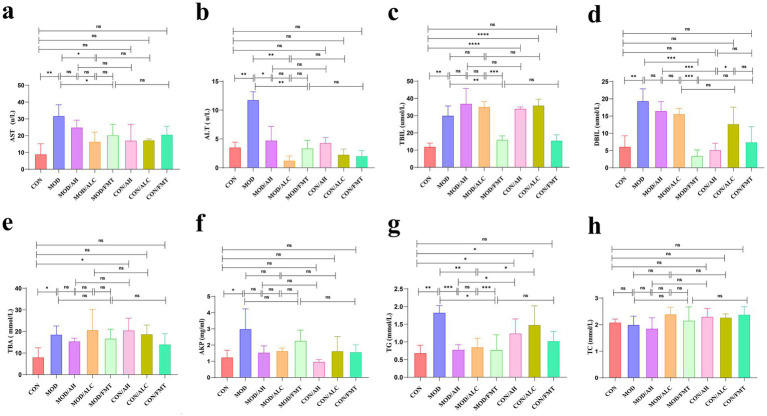
Serum biochemical indicators of liver function. **(a–h)** Changes in related serological parameters. *n* ≥ 12 per group. Data are presented as mean ± SD. Statistical analysis among multiple groups was performed using one-way ANOVA or Kruskal–Wallis test as appropriate, followed by post hoc multiple comparisons. * *p* < 0.05, ** *p* < 0.01, *** *p* < 0.001.

To systematically evaluate the effects of FMT from different donor sources on ALD, we quantitatively analyzed serum biochemical markers of liver function in mice from each experimental group (Figure 5). Compared with the CON group, mice in the MOD exhibited significantly elevated serum markers associated with hepatocellular injury and bile metabolism, including AST (*p* = 0.0058; Figure 5a), ALT (*p* = 0.0036; Figure 5b), TBIL (*p* = 0.0031; Figure 5c), DBIL (*p* = 0.0014; Figure 5d), TBA (*p* = 0.0244; Figure 5e), AKP (*p* = 0.0357; Figure 5f), and TG (*p* = 0.0033; Figure 5g). These findings indicate that alcohol exposure induced marked hepatocellular damage and bile metabolic dysfunction.

Following FMT intervention, FMT derived from healthy donors significantly improved multiple serum biochemical parameters in the alcohol model context, including AST (*p* = 0.047; Figure 5a), ALT (*p* = 0.0067; Figure 5b), TBIL (*p* = 0.0093; Figure 5c), DBIL (*p* = 0.0002; Figure 5d), TBA (*p* = 0.0353; Figure 5e), and TG (*p* = 0.0220; Figure 5g), demonstrating a stable protective effect in attenuating hepatocellular injury and bile metabolic abnormalities.

In contrast, FMT derived from patients with AH failed to confer improvement and instead further increased ALT (*p* = 0.0241; Figure 5b) and TG (*p* < 0.0001; Figure 5g) levels in alcohol-fed mice, along with significantly elevated DBIL and TBA levels. These adverse alterations were also observed in mice under the control diet background (all *p* < 0.0001; Figures 5c–e), suggesting that AH-derived microbiota may exacerbate liver dysfunction across different host metabolic contexts. FMT from patients with AC resulted in moderate reductions in AST and ALT levels (both *p* < 0.05; Figures 5a,b). However, this was accompanied by increased TBIL, TBA, and TG levels (all *p* < 0.05; Figures 5c,e,f) in both MOD and CON mice, failing to demonstrate a consistent overall biochemical improvement. In addition, TC showed no significant differences among groups (Figure 5h).

In summary, comprehensive analysis of serum biochemical indicators indicates that only FMT derived from healthy donors consistently ameliorated hepatocellular injury and bile metabolic disturbances in the ALD model. In contrast, microbiota transplantation from patients exhibited limited or even detrimental biochemical effects, providing a functional basis for subsequent investigations into donor-dependent metabolic and immunological mechanisms underlying FMT efficacy.

#### 3.3.3 Healthy-donor FMT modulates systemic and local inflammation

**Figure 6:**
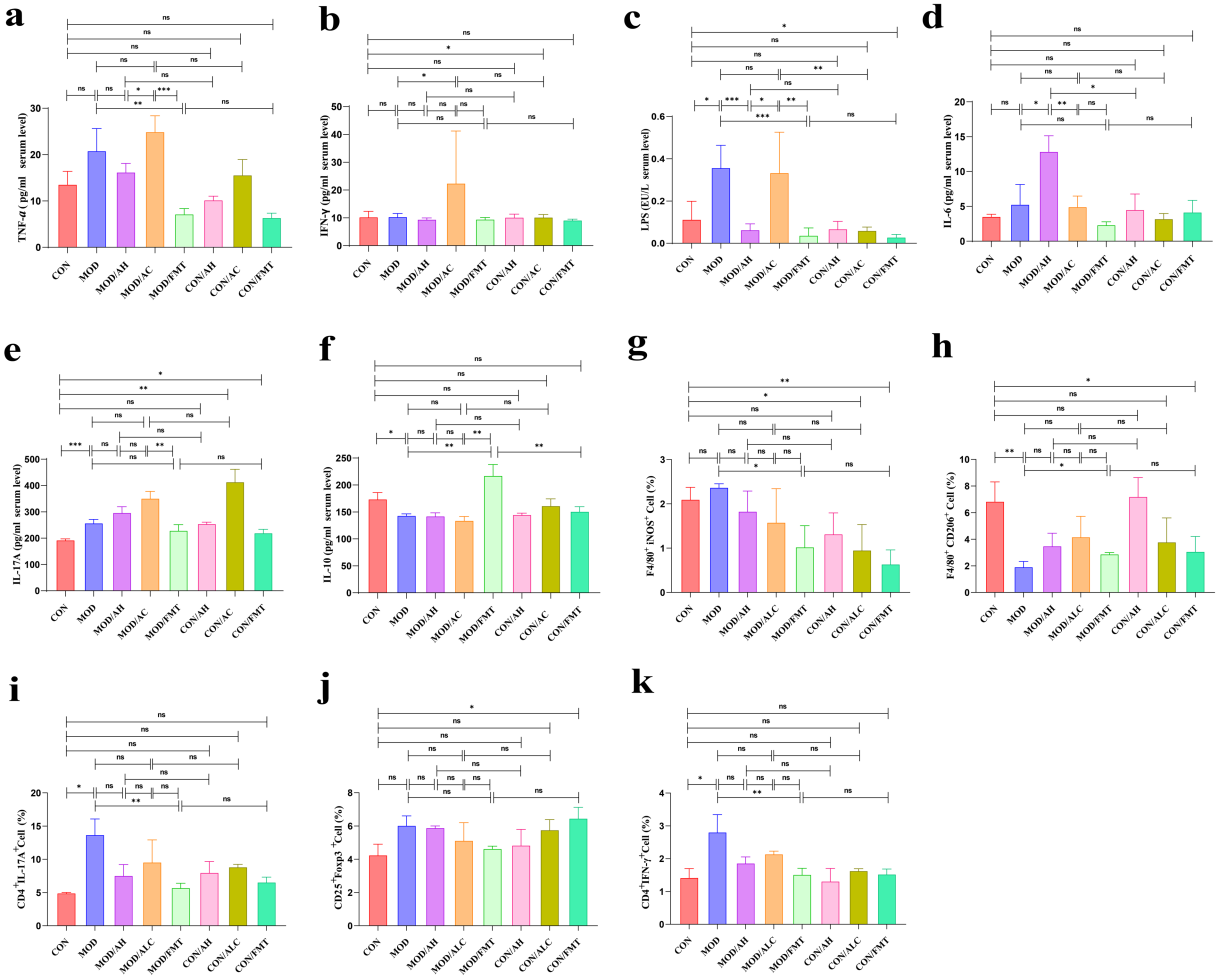
Expression of inflammatory cytokines in serum, liver, and intestinal tissues. **(a–f)** Expression of inflammatory factors in serum. **(g)** Hepatic M1 macrophages (F4/80^+^CD11b^+^iNOS^+^). **(h)** Hepatic M2 macrophages (F4/80^+^CD11b^+^CD206^+^). **(i)** Splenic Th17 cells (CD4^+^IL-17A^+^). **(j)** Splenic Tregs (CD4^+^CD25^+^Foxp3^+^). **(k)** IFN-*γ* producing T cells (CD4^+^IFN-γ^+^). *n* ≥ 4 per group. Data are presented as mean ± SD. Statistical comparisons among multiple groups were conducted using one-way ANOVA or Kruskal–Wallis test as appropriate, followed by post hoc multiple-comparison analysis. * *p* < 0.05, ** *p* < 0.01, *** *p* < 0.001.

We next assessed cytokines and macrophage polarization (Figure 6). MOD mice showed higher plasma TNF-*α* (*p* = 0.0924), LPS (*p* = 0.0389), and IL-17A (*p* = 0.0006) and lower IL-10 (*p* = 0.0124) than controls. Healthy-donor FMT lowered TNF-α (*p* = 0.0095), LPS (*p* = 0.0002) and increased IL-10 (*p* = 0.0032) (Figures 6a–f). Similar anti-inflammatory effects were observed in liver and intestinal tissues (Supplementary Figures S5a–l), where healthy FMT reduced TNF-α, IL-6, IL-17A, and LPS and restored IL-10. AH-FMT and AC-FMT either blunted or reversed these benefits, often raising IL-6 or LPS. Flow cytometry (Figures 6g–k; Supplementary Figures S6a–c) revealed that healthy-donor FMT suppressed pro-inflammatory M1 macrophages in both MOD (*p* = 0.0108) and control (*p* = 0.0051) mice and rescued the disease-associated depletion of anti-inflammatory M2 macrophages (*p* = 0.0316). For T-cell subsets, healthy FMT lowered splenic Th17 (*p* = 0.0031) and IFN-*γ* (*p* = 0.0085) and modestly increased regulatory T cells in controls (*p* = 0.0433). Overall, healthy-donor FMT exerts broad immunomodulatory effects, dampening pro-inflammatory signals and promoting anti-inflammatory responses.

#### 3.3.4 Healthy-donor FMT restores intestinal barrier integrity and blood flow

**Figure 7:**
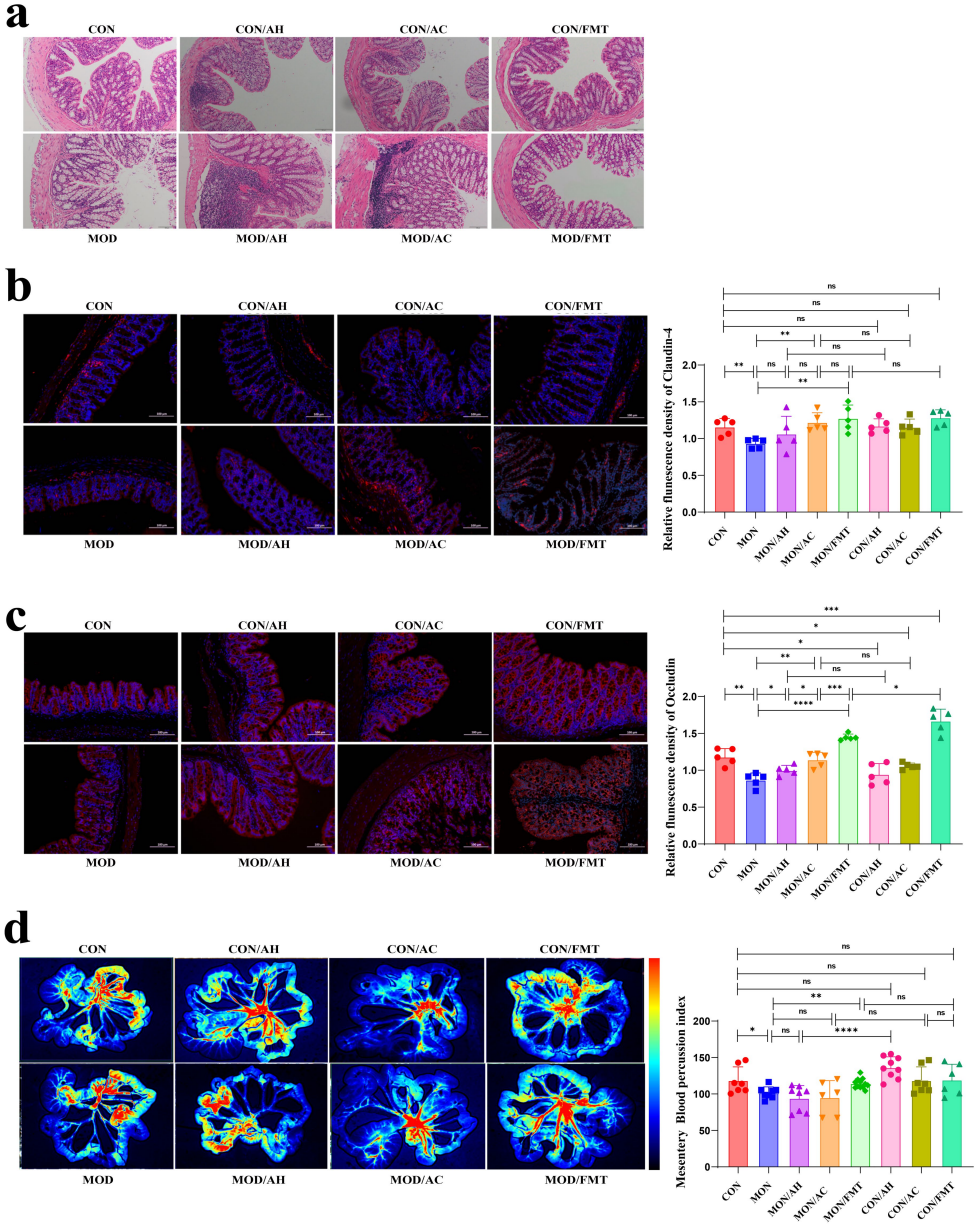
FMT improves intestinal barrier integrity and mesenteric circulation. **(a)** Colonic histology (H&E staining). **(b,c)** Immunofluorescence staining of Claudin-4 and Occludin in colonic tissue and semi-quantitative densitometric analysis of Claudin-4 and Occludin expression. **(d)** Mesenteric blood flow detected by laser speckle imaging and quantitative analysis of mesenteric perfusion. *n* ≥ 4 per group. Data are presented as mean ± SD. Statistical comparisons among multiple groups were conducted using one-way ANOVA or Kruskal–Wallis test as appropriate, followed by post hoc multiple-comparison analysis. * *p* < 0.05, ** *p* < 0.01, *** *p* < 0.001.

Colon histology showed widened villus gaps and shallow crypts in MOD mice, changes improved by healthy-donor FMT (*p* < 0.05), but worsened by AH-FMT or AC-FMT (Figure 7a). Furthermore, immunofluorescence confirmed reduced occludin (*p* = 0.0015) and claudin-4 (*p* = 0.0053) expression in MOD mice (Figure 7b). Healthy FMT markedly increased occludin (*p* < 0.0001) and claudin-4 (*p* < 0.001), whereas patient-derived FMT lowered occludin without affecting claudin-4 (Figure 7c). Consistently, mesenteric laser-speckle imaging demonstrated diminished intestinal perfusion in MOD mice (*p* = 0.0377); healthy-donor FMT restored blood flow (*p* = 0.0015), while AH-FMT or AC-FMT had no benefit (Figure 7d). Thus, these findings indicate that healthy-donor FMT strengthens intestinal tight junctions and improves mucosal perfusion, thereby repairing the gut barrier.

### 3.4 Healthy-donor FMT restores microbial homeostasis in ALD mice

**Figure 8:**
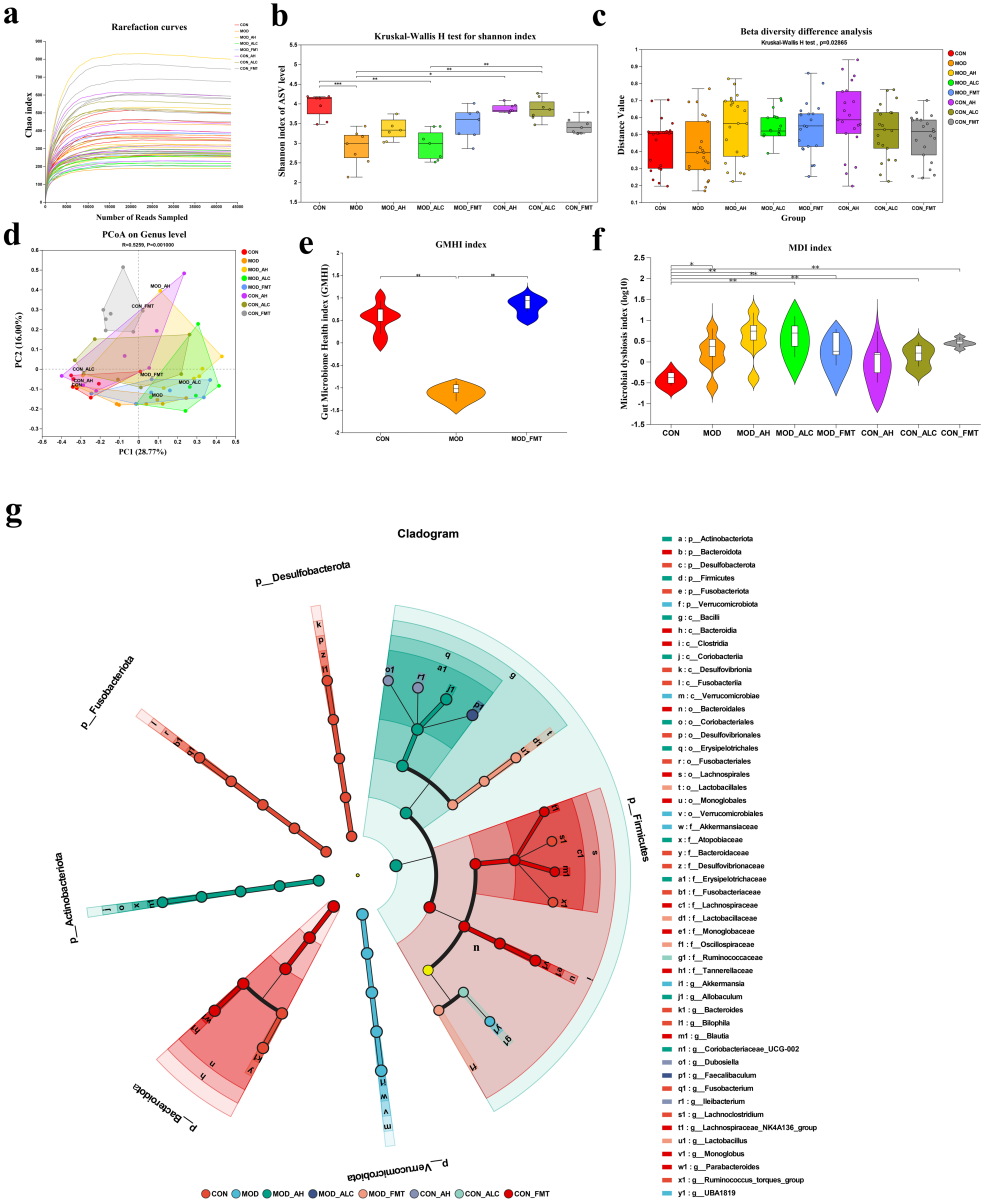
Analysis of gut microbial diversity and community structure. **(a)** Rarefaction curve. **(b)** Shannon index comparison. **(c)** ASV-level beta distance index (*p* = 0.02865). **(d)** PCoA analysis. **(e)** Gut microbiome health index (GMHI). **(f)** Microbial dysbiosis index (MDI). **(g)** LEfSe analysis of multilevel species differences. *n* ≥ 5 per group. Data are presented as mean ± SD. Statistical comparisons among multiple groups were conducted using one-way ANOVA or Kruskal–Wallis test as appropriate, followed by post hoc multiple-comparison analysis. * *p* < 0.05, ** *p* < 0.01, *** *p* < 0.001.

To systematically evaluate the impact of FMT from different donor sources on the gut microbiota of mice, 16S rRNA gene sequencing was performed on fecal samples from all groups (Figure 8). Rarefaction curves reached a plateau across all samples, indicating that sequencing depth was sufficient to capture the majority of microbial species and that the dataset was adequate for subsequent diversity and community structure analyses (Figure 8a). Alpha diversity analysis revealed a significant reduction in the Shannon index in MOD mice compared with controls (*p* < 0.001), indicating that ethanol exposure markedly impaired gut microbial diversity. Healthy-donor FMT significantly increased the Shannon diversity index in MOD mice, whereas AH- and AC-derived FMT induced only modest, non-significant increases (Figure 8b). At the community structure level, beta diversity analysis based on amplicon sequence variants (ASVs) demonstrated significant separation among treatment groups (PERMANOVA, *p* = 0.02865). Principal coordinates analysis (PCoA) further confirmed that the microbial composition of MOD mice was distinctly separated from that of controls, whereas healthy-donor FMT shifted the microbial community structure toward the control cluster. In contrast, the AH-FMT and AC-FMT groups largely overlapped with the MOD group, indicating limited restoration of microbial composition (Figures 8c,d). To further evaluate microbial functional status, the Gut Microbiome Health Index (GMHI) and Microbial Dysbiosis Index (MDI) were calculated. GMHI was significantly reduced in MOD mice (*p* = 0.0022) and was partially restored following healthy-donor FMT (*p* = 0.041) (Figure 8e). Consistently, MDI was markedly elevated in MOD mice (*p* = 0.0106) and further increased after AH-FMT and AC-FMT intervention (both *p* < 0.03), whereas healthy-donor FMT significantly reduced MDI (*p* = 0.003) (Figure 8f).

For further analysis of community composition, Venn (Supplementary Figures S7a–c) and bar-plot (Supplementary Figures S7d–f) analyses demonstrated significant group differences at phylum, genus, and species levels. Notable phylum-level shifts included Firmicutes (*p* = 0.0026) and Bacteroidota (*p* = 0.028). Genera enriched after healthy FMT included Akkermansia (*p* = 3.0 × 10^−5^) and SCFA producing probiotics including Allobaculum and Faecalibaculum (both *p* < 0.001). Additionally, LEfSe analysis (LDA > 3.5) (Figure 8g) identified Akkermansia as a dominant candidate in MOD-FMT and CON-FMT mice, consistent with improved barrier function and anti-inflammatory activity. In contrast, AH- and AC-FMT promoted expansion of taxa linked to gut dysbiosis. Taken together, these results demonstrate that healthy-donor FMT re-establishes balanced microbial diversity and composition, whereas patient-derived FMT worsens dysbiosis.

### 3.5 Healthy-donor FMT elevates SCFAs

**Figure 9:**
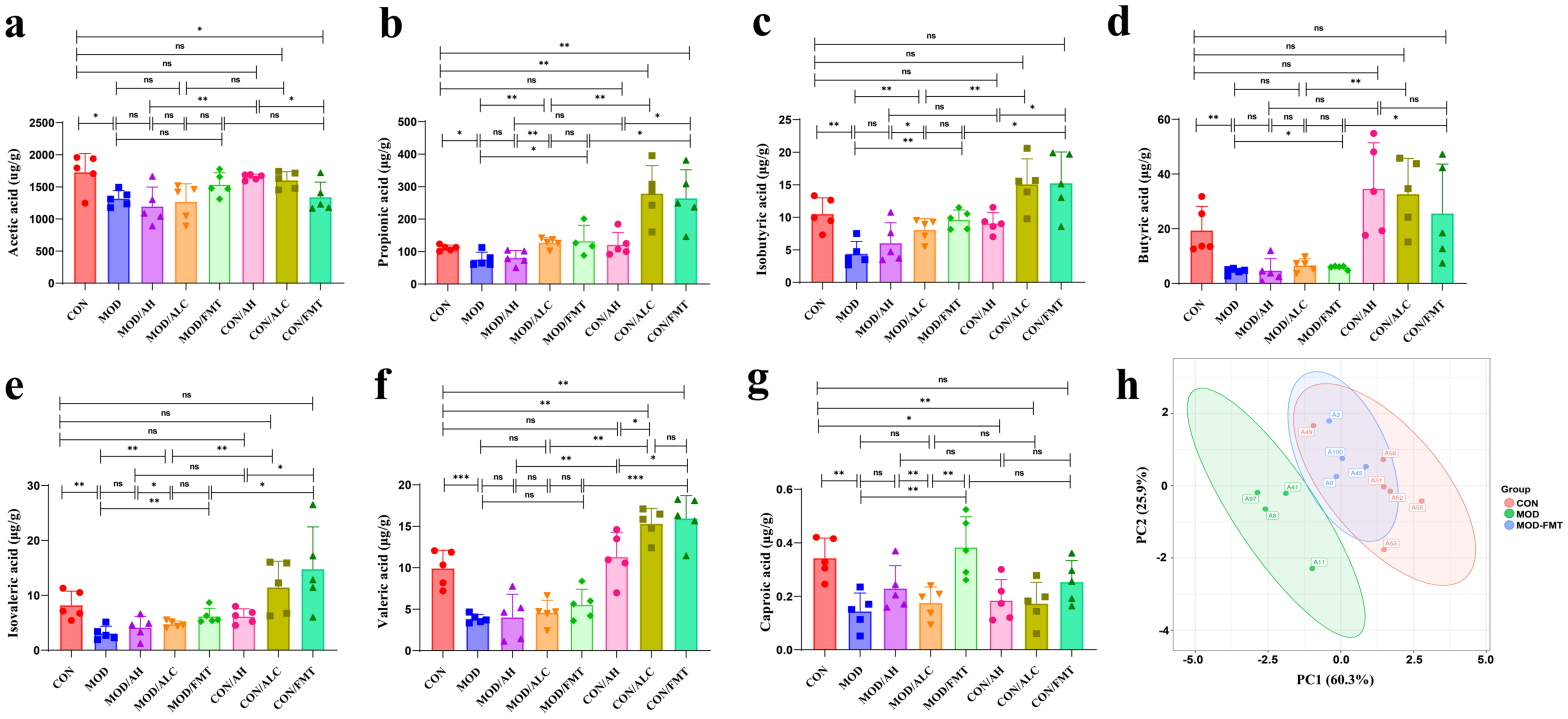
FMT enhances SCFA production in the gut. **(a–g)** Concentrations of acetic, propionic, isobutyric, butyric, isovaleric, valeric, and caproic acids. **(h)** Principal component analysis (PCA). *n* ≥ 5 per group. Data are presented as mean ± SD. Statistical comparisons among multiple groups were conducted using one-way ANOVA or Kruskal–Wallis test as appropriate, followed by post hoc multiple-comparison analysis. * *p* < 0.05, ** *p* < 0.01, *** *p* < 0.001.

To evaluate the effects of FMT from different donor sources on intestinal metabolic function, SCFAs in mouse feces were quantified using gas chromatography–mass spectrometry (GC–MS) (Figure 9). Representative chromatograms demonstrated clear peak separation of major SCFAs across groups. QC samples exhibited acceptable RSDs, indicating good analytical stability and reproducibility of the detection platform (Supplementary Figures S8a,b). Hierarchical clustering heatmap analysis revealed a distinct metabolic profile in MOD mice compared with controls, characterized by a global downward trend in SCFA abundance. In contrast, the metabolic profile of the healthy-donor FMT group shifted toward that of controls, suggesting partial normalization of intestinal metabolic status (Supplementary Figure S8c).

Compared with controls, MOD mice exhibited significantly reduced levels of multiple SCFAs, including acetate (*p* = 0.019), propionate (*p* = 0.011), isobutyrate (*p* = 0.002), butyrate (*p* = 0.005), isovalerate (*p* = 0.004), valerate (*p* = 0.0003), and hexanoate (*p* = 0.002), indicating ethanol-induced impairment of gut metabolic function. Healthy-donor FMT significantly increased several SCFAs, particularly propionate (*p* = 0.048), isobutyrate (*p* = 0.0011), butyrate (*p* = 0.035), isovalerate (*p* = 0.0062), and hexanoate (*p* = 0.0037). Acetate and valerate also showed upward trends, suggesting that healthy-donor FMT effectively restores SCFA biosynthetic capacity. In contrast, AH-derived FMT exerted limited effects on SCFA restoration. AC-derived FMT produced inconsistent metabolic changes: although some SCFAs showed mild increases, hexanoate levels were significantly reduced (*p* = 0.0083), suggesting potential adverse metabolic effects associated with patient-derived microbiota (Figures 9a–g).

Principal component analysis (PCA) further supported these findings at the global metabolic level. Samples from the healthy-donor FMT group were clearly separated from MOD mice in PCA space and clustered toward the control group, whereas AH-FMT and AC-FMT samples largely overlapped with the MOD cluster (Figure 9h). Collectively, these data indicate that only healthy-donor FMT effectively corrects ethanol-induced disturbances in SCFA metabolism.

### 3.6 Healthy-donor FMT reprograms fecal metabolome

**Figure 10:**
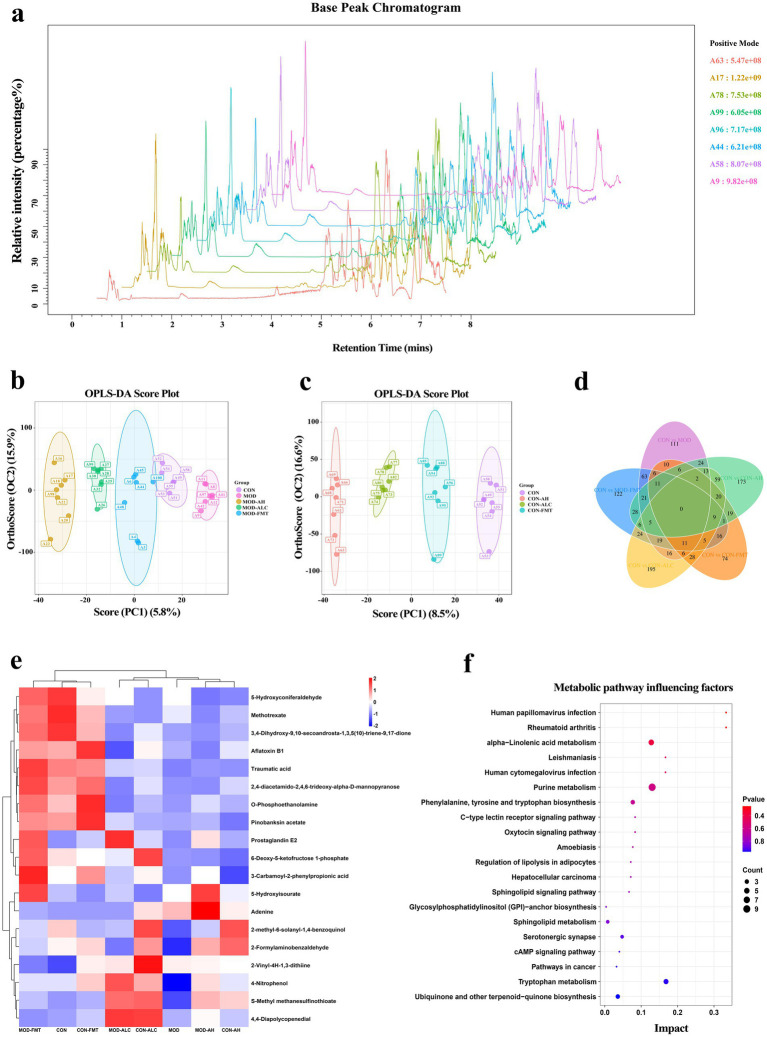
Metabolomic profiling of fecal samples. **(a)** Base peak chromatogram. **(b,c)** OPLS-DA score plots. **(d)** Venn diagram of secondary differential metabolites. **(e)** Heatmap of metabolite abundance. **(f)** Bubble chart illustrating key metabolic pathways and their impact scores. *n* ≥ 5 per group.

To systematically evaluate the impact of FMT on the intestinal metabolic landscape, untargeted metabolomic profiling of mouse fecal samples was performed using liquid chromatography–tandem mass spectrometry (LC–MS/MS) (Figure 10). Base peak chromatograms demonstrated consistent peak distribution and stable signal intensity across groups. The RSD of QC samples was below 30%, indicating acceptable analytical stability and overall data reliability (Figure 10a). At the global metabolic level, orthogonal partial least squares–discriminant analysis (OPLS-DA) revealed clear separation among the control, MOD, and healthy-donor FMT groups in the score plots, suggesting that ethanol exposure markedly reshaped the fecal metabolomic profile, whereas healthy-donor FMT induced substantial metabolic reprogramming (Figures 10b,c). Differential metabolite analysis further demonstrated extensive metabolic alterations in MOD mice compared with controls. Healthy-donor FMT significantly remodeled these abnormal metabolic signatures. Venn diagram analysis revealed both shared and FMT-specific sets of differentially regulated metabolites across groups (Figure 10d). Volcano plots illustrated multiple significantly upregulated and downregulated metabolites (Supplementary Figures S9a–k), while hierarchical clustering heatmap analysis showed that the overall metabolite abundance pattern in the healthy-donor FMT group shifted toward that of controls, indicating partial metabolic restoration (Figure 10e). At the pathway level, enrichment analysis and bubble plots identified several significantly affected metabolic pathways following healthy-donor FMT, particularly ALA metabolism, purine metabolism, and phenylalanine metabolism (Figure 10f). These pathways are closely associated with energy metabolism, amino acid metabolism, and the biosynthesis of microbially derived bioactive metabolites, highlighting the substantial role of FMT in reshaping the intestinal metabolic network. Collectively, these findings indicate that healthy-donor FMT induces broad reprogramming of the fecal metabolome and partially restores ethanol-disrupted metabolic homeostasis.

### 3.7 Correlations between microbiota, metabolites, and host responses

**Figure 11:**
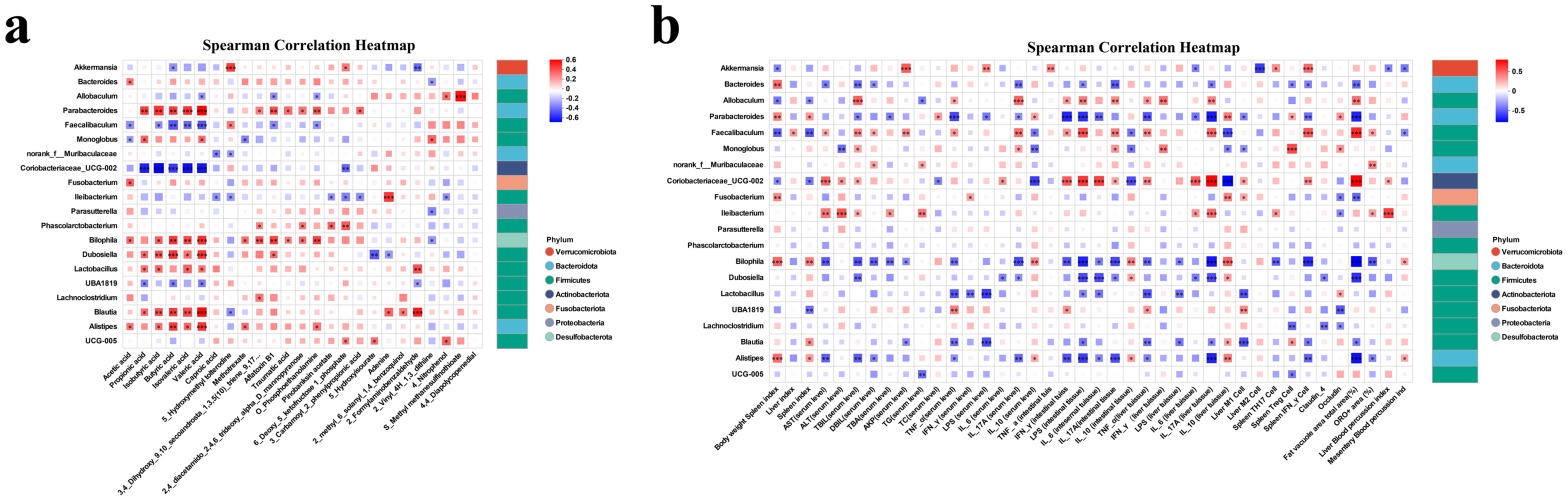
Correlation analysis between gut microbial taxa, metabolites, and host responses. **(a)** Spearman correlation heatmap illustrating the associations between the relative abundance of gut microbial genera and fecal metabolites. Color intensity represents the Spearman correlation coefficient (*ρ*), with red indicating positive correlations and blue indicating negative correlations. Significant correlations are indicated by asterisks. **(b)** Spearman correlation heatmap showing correlations between gut microbial genera and host physiological, biochemical, and immunological parameters, including body weight, organ indices, serum liver function markers, inflammatory cytokines, and endotoxin levels. Only statistically significant correlations are highlighted. * *p* < 0.05, ** *p* < 0.01, *** *p* < 0.001.

To elucidate the interactions among gut microbiota, metabolic profiles, and host physiological responses, Spearman correlation analyses were performed (Figures 11a,b).

As shown in Figure 11a, multiple bacterial genera exhibited significant associations with diverse metabolites. Genera such as Lachnoclostridium, Blautia, Faecalibaculum, and Parabacteroides displayed strong positive correlations with SCFAs including acetic, propionic, and butyric acids, suggesting their potential contribution to SCFA production and intestinal homeostasis. In contrast, Akkermansia and Allobaculum were negatively correlated with several amino acid derivatives and nitrogenous metabolites, indicating a possible regulatory role in host amino acid and nitrogen metabolism. Furthermore, Bilophila and Dubosiella were positively associated with lipid- and bile acid-related metabolites, while Parasutterella showed negative correlations with carbohydrate metabolites. These patterns suggest that specific microbial taxa may shape host metabolic pathways and energy utilization.

**Figure 12:**
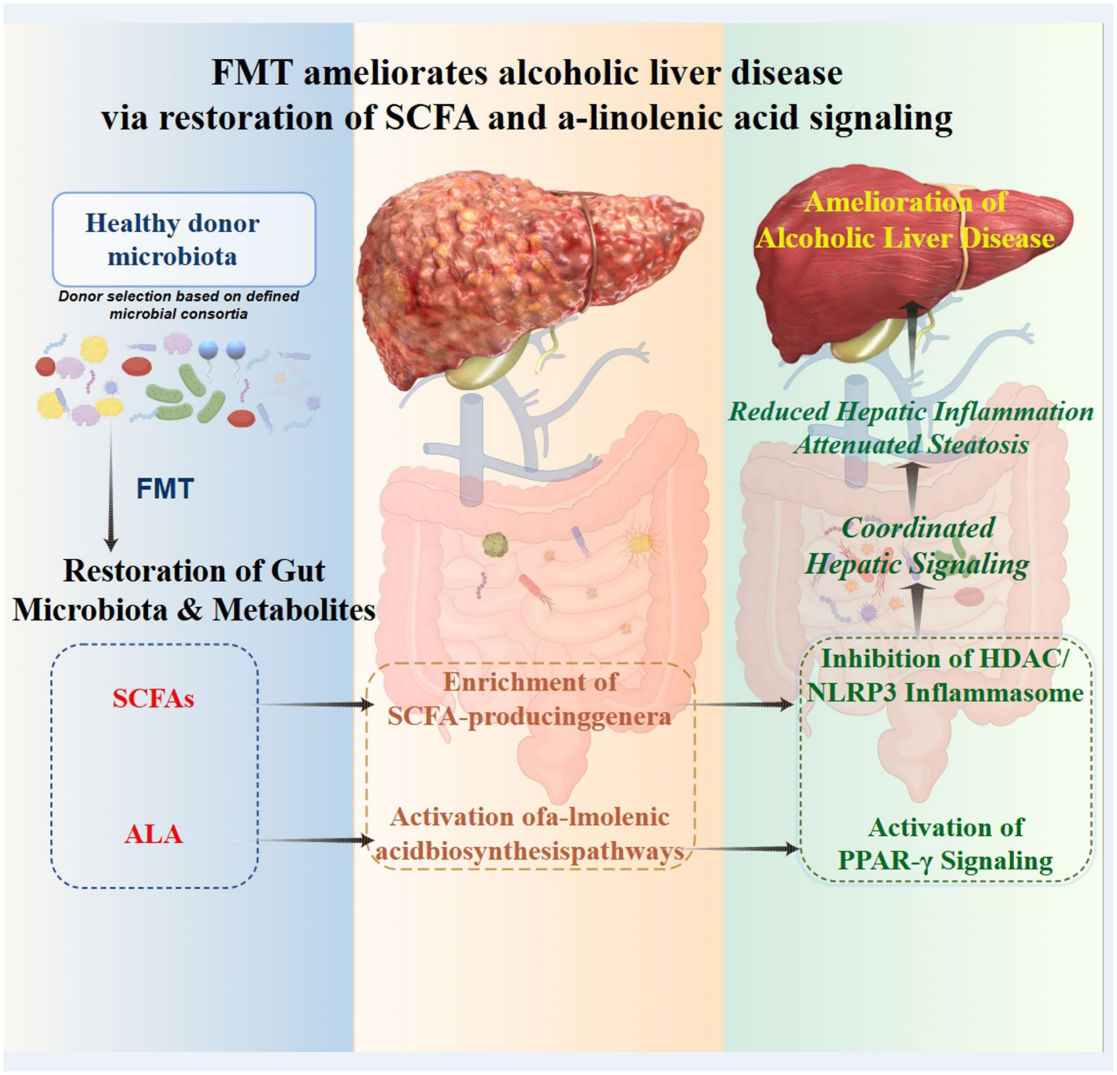
Mechanism model diagram.

To further explore host–microbe interactions, correlations between bacterial genera and host biochemical as well as immunological parameters were analyzed (Figure 11b). Genera such as Faecalibaculum, Lachnoclostridium, and Blautia were positively correlated with favorable host indicators including body weight, spleen index, and the anti-inflammatory cytokine IL-10, implying their potential protective roles in maintaining hepatic and systemic homeostasis. Conversely, Akkermansia, Bilophila, and Dubosiella were negatively correlated with serum liver injury markers (ALT, AST, and TBA) and pro-inflammatory cytokines (IL-6, TNF-*α*, IFN-*γ*, and IL-17A), indicating possible involvement in inflammation and hepatocellular injury. Additionally, Lactobacillus and Parabacteroides exhibited negative correlations with LPS levels in both serum and intestinal tissues, suggesting their role in preserving gut barrier integrity and reducing endotoxemia (Figure 12).

Taken together, these findings reveal a close and multifaceted relationship between the gut microbiota, host metabolism, and immune responses. Specific bacterial taxa may modulate hepatic and intestinal homeostasis by influencing metabolic reprogramming and inflammatory signaling, thereby shaping disease outcomes and therapeutic responses.

These integrative correlations between gut microbiota, metabolites, and host responses suggest that microbial dysbiosis may exert systemic effects through metabolic and immune pathways. Therefore, further discussion focuses on the potential mechanisms by which specific microbial taxa and their metabolites modulate hepatic inflammation and intestinal homeostasis.

## 4 Discussion

### 4.1 Progressive gut dysbiosis as an important ecological characteristic of ALD

Our study delineates a dynamic trajectory of gut microbial collapse that underpins the progression of ALD. Consistent with previous study, AH stage is primarily characterized by significant remodeling of the microbiota composition (e.g., relative increase in pathogenic bacteria) (Philips et al., 2024). In the AC stage, it further progresses to an ecological collapse characterized by a decline in overall α-diversity, loss of key commensal bacteria (e.g., *Blautia, Faecalibacterium*), and enrichment of pathogenic bacteria (Baltazar-Diaz et al., 2022). A similar microbial evolutionary trajectory of “from imbalance to collapse” has been systematically described in related studies and is considered a universal feature of ALD progression (Kanagalingam and Bajaj, 2024). Functional analyses further revealed increased VF gene abundance in AH and compensatory enrichment of antibiotic resistance genes in AC (Figure 4), reinforcing the concept that microbial perturbation is an active pathogenic driver rather than a passive consequence of disease progression (Cassard and Ciocan, 2018; Xiang et al., 2024).

### 4.2 FMT exerts therapeutic efficacy via donor-dependent microbial restoration

We demonstrate that the ameliorative effect of FMT on ALD exhibits significant donor dependency. FMT from healthy donors persistently improved liver histopathology, biochemical indicators, and inflammatory status in ALD model mice, whereas FMT from diseased donors failed to produce similar effects. This phenomenon suggests that FMT is not merely a “microbiota replacement” but rather represents a process of functional microbial community reconstruction. Notably, we systematically examined changes in the gut microbiota structure after FMT in recipient mice and observed stable and significant community remodeling, indicating effective colonization of the transplanted microbiota against a background of antibiotic pretreatment. These results are consistent with previous animal studies showing that, in mouse models pretreated with standardized antibiotics, FMT can achieve reconstruction and relatively stable colonization of the donor microbiota, with significant changes observed in the gut microbiota structure and host metabolism/phenotype of the recipients (Taur et al., 2018; Lesniak et al., 2022; Yang et al., 2025). These findings suggest that FMT can produce stable and detectable biological effects in the model used in this study.

### 4.3 Synergistic restoration of key metabolic functions by SCFAs and ALA

At the metabolic level, FMT from healthy donors enhanced the SCFA-producing capacity of the recipient gut microbiota, a change closely associated with the observed alleviation of inflammation and improvement in immune homeostasis. Notably, the restoration of butyrate levels was negatively correlated with a reduction in the proportion of pro-inflammatory M1 macrophages and Th17 responses, accompanied by an upregulation in the proportion of regulatory T cells. These results are consistent with previous studies showing that butyrate can participate in the regulation of macrophage polarization and T-cell responses by inhibiting histone deacetylases (Wang et al., 2026; Taylor et al., 2014). In addition to SCFAs, this study further found that ALA-related metabolic pathways were significantly enhanced after FMT. The changes in this pathway are consistent with previous reports that ALA and its derivatives are closely associated with the regulation of lipid metabolism, alleviation of oxidative stress, and modulation of inflammatory responses, and demonstrate regulatory potential in various metabolic disorders and inflammatory models (Takic et al., 2024; Liu et al., 2025).

### 4.4 Translational implications and outlook

The findings of this study provide a critical translational perspective and scientific basis for the application of FMT in the treatment of ALD. Currently, experimental and early clinical evidence has shown that FMT can prevent or ameliorate alcohol-related liver injury, providing preliminary support for its clinical feasibility. The value of this study lies in its in-depth exploration of the possible core mechanisms underlying the efficacy of FMT by integrating multi-omics analyses, beyond the mere observation of whether FMT is effective. Our results indicate that the efficacy of FMT from healthy donors is closely related to its ability to systematically reconstruct specific key microbial metabolic functions, particularly the biosynthesis of SCFAs and ALA. The restoration of these two metabolites is directly associated with gut ecological repair, alleviation of liver inflammation, and improvement in immune homeostasis. This provides new mechanistic insights into FMT treatment for ALD, shifting the focus from “microbiota transplantation” to “functional remodeling.” It implies that future microbiome interventions should not merely aim for “successful transplantation” or changes in microbiota composition but should focus on evaluating and ensuring the functional restoration of these key beneficial metabolic pathways. This provides a direction for the development of more targeted next-generation live biotherapeutic products or synbiotics based on specific metabolites or their precursor bacteria.

## 5 Limitations

All animal experiments in this study were conducted using female mice, whereas the majority of clinical patients with ALD are male. Gender factors may indirectly influence gut microbiota structure and host metabolic phenotype through differences in hormone levels, basal metabolic background, and immune responses. Therefore, the generalizability and weight of the “microbiota-metabolism-immunity” axis elucidated in this study based on a female model need to be systematically validated in future studies by establishing parallel gender cohorts in male patients. Additionally, through multi-omics association analysis, this study proposed the hypothesis of synergistic effects between the SCFA and ALA pathways and observed corresponding improvements in immune phenotypes. However, the definitive *in vivo* proof that these two metabolites are the sole and essential downstream mediators of FMT efficacy has not been established through receptor antagonists, gene knockout models, or direct metabolite supplementation experiments. Therefore, the current mechanistic associations provide strong suggestive evidence, and the exact causal chain remains to be confirmed by subsequent functional studies.

## 6 Future directions

To address these limitations and facilitate clinical translation, we propose:

(1) In animal models, use GPR43, GPR120, or PPAR*α* gene knockout mice or administer specific receptor antagonists to validate the causal role of SCFA and ALA signaling pathways in mediating the efficacy of FMT.
(2) Develop pre-screened microbial communities with high SCFA/ALA production capacity.
(3) Conduct longitudinal studies to assess the durability of metabolic reconstruction mediated by FMT.

## 7 Conclusion

This study confirms that FMT from healthy donors effectively reconstructs gut ecology in a donor-dependent manner. The core mechanism may be closely related to the synergistic restoration of two key microbial metabolic axes, SCFAs and ALA, thereby improving intestinal barrier function, liver inflammation, and immune imbalance through multiple targets. These findings not only provide new functional mechanistic insights beyond microbiota composition for FMT treatment of ALD but also support the use of microbial metabolic function (not merely species composition) as a core target for the development of microecological therapies for ALD.

## Data Availability

The datasets presented in this study can be found in online repositories. The names of the repository/repositories and accession number(s) can be found in the article/Supplementary material.

## Supplementary material

SUPPLEMENTARY FIGURE S1 Survival curves of mice in the AH and AC groups.

SUPPLEMENTARY FIGURE S2 Differential taxonomic features across different stages of alcohol-related liver disease. **(a)** LEfSe cladogram analysis: differentially enriched taxa among groups were identified using LEfSe with an LDA score threshold >3.5. The cladogram illustrates the phylogenetic relationships of significantly enriched taxa and their corresponding group associations. **(b)** LDA scores of discriminative taxa identified by LEfSe: representative taxa that significantly distinguished AH, AC, and healthy control groups are displayed along with their corresponding LDA scores, quantifying their relative contributions to intergroup discrimination. **(c–e)** Abundance comparisons of representative differential taxa: Selected differentially abundant taxa are presented at the phylum **(c)**, genus **(d)**, and species **(e)** levels, showing their mean relative abundances across groups. Intergroup differences were assessed using the Kruskal–Wallis test. Bar plots represent mean values, and error bars indicate the standard error of the mean (SEM).

SUPPLEMENTARY FIGURE S3 Functional supportive analysis of the gut microbiota across different stages of ALD. **(a)** LEfSe analysis based on the Comprehensive Antibiotic Resistance Database (CARD) (LDA >3). Differentially enriched antibiotic resistance genes among groups were identified using LEfSe with an LDA score threshold >3, based on CARD annotations. **(b)** Weighted Gene Co-expression Network Analysis (WGCNA). WGCNA was performed to identify co-expression modules of microbial genes and to explore their associations with clinical phenotypes across different stages of ALD.

SUPPLEMENTARY FIGURE S4 Effects of FMT from different donor sources on hepatic histological architecture and microcirculatory perfusion in ALD mice. **(a)** H&E staining. Representative liver sections showing hepatocellular morphological alterations across different groups. **(b)** Oil Red O staining. Visualization of hepatic lipid accumulation to assess the degree of steatosis. **(c)** Hepatic perfusion analysis. Laser speckle imaging was used to evaluate the effects of different FMT interventions on hepatic microcirculatory perfusion. Scale bar = 100 μm.

SUPPLEMENTARY FIGURE S5 Expression of inflammatory cytokines in liver and intestinal tissues. **(a–l)** Expression levels of inflammatory factors in liver **(a–f)** and intestinal **(g–l)** tissues following FMT. *n* ≥ 4 per group. Data are presented as mean ± SD. Statistical comparisons among multiple groups were conducted using one-way ANOVA or Kruskal–Wallis test as appropriate, followed by post hoc multiple-comparison analysis. * *P* < 0.05, ** *P* < 0.01, *** *P* < 0.001.

SUPPLEMENTARY FIGURE S6 Flow cytometric analysis of immune cell populations. **(a)** Hepatic M1 macrophages (F4/80⁺CD11b⁺iNOS⁺) and M2 macrophages (F4/80⁺CD11b⁺CD206⁺). **(b)** Splenic Th17 cells (CD4⁺IL-17A⁺) and Tregs (CD4⁺CD25⁺Foxp3⁺). **(c)** IFN-γ–producing T cells (CD4⁺IFN-γ⁺). *n* ≥ 4 per group.

SUPPLEMENTARY FIGURE S7 Taxonomic differences in gut microbiota composition among groups. **(a–c)** Venn diagrams showing shared and unique ASVs among different groups. **(d–f)** Relative abundance of gut microbial taxa at the phylum, genus, and species levels. *n* ≥ 5 per group. Data are presented as mean ± SD. Statistical comparisons among multiple groups were conducted using one-way ANOVA or Kruskal–Wallis test as appropriate, followed by post hoc multiple-comparison analysis. * *P* < 0.05, ** *P* < 0.01, *** *P* < 0.001.

SUPPLEMENTARY FIGURE S8 Quality control and clustering analysis of fecal SCFAs. **(a)** Representative GC–MS chromatograms of fecal SCFAs. **(b)** RSD of quality control samples. **(c)** Cluster heatmap of fecal SCFA profiles across groups. *n* ≥ 5 per group.

SUPPLEMENTARY FIGURE S9 Differential metabolite analysis of fecal samples. **(a–k)** Volcano plots showing significantly altered metabolites among groups based on non-targeted LC–MS/MS analysis. *n* ≥ 5 per group.

## Glossary

ALD: alcohol-associated liver disease
FMT: fecal microbiota transplantation
SCFA: short-chain fatty acid
ALA: α-linolenic acid
AH: alcoholic hepatitis
AC: alcohol-associated cirrhosis
AST: aspartate aminotransferase
ALT: alanine aminotransferase
AKP: alkaline phosphatase
TBIL: total bilirubin
DBIL: direct bilirubin
TBA: total bile acids
TG: triglycerides
ELISA: enzyme-linked immunosorbent assay
LPS: lipopolysaccharide
QC: quality control
RSD: relative standard deviation
FDR: false discovery rate
VF: virulence factor
VFDB: Virulence Factor Database
COG: clusters of orthologous groups
CAZy: carbohydrate-active enzyme
WGCNA: weighted gene co-expression network analysis
GMHI: Gut Microbiome Health Index
MDI: Microbial Dysbiosis Index
GC–MS: gas chromatography–mass spectrometry
PCA: principal component analysis
LC–MS/MS: liquid chromatography–tandem mass spectrometry
OPLS-DA: orthogonal partial least squares–discriminant analysis
SEM: standard error of the mean
LEfSe: linear discriminant analysis effect size
H&E: Hematoxylin and eosin

## References

[ref1] Baltazar-Diaz T. A. González-Hernández L. A. Aldana-Ledesma J. M. Peña-Rodríguez M. Vega-Magaña A. N. Zepeda-Morales A. S. M. . (2022). *Escherichia*/*Shigella*, SCFAs, and metabolic pathways-the triad that orchestrates intestinal dysbiosis in patients with decompensated alcoholic cirrhosis from Western Mexico. Microorganisms 10:1231. doi: 10.3390/microorganisms10061231, 35744749 PMC9229093

[ref2] Cassard A. M. Ciocan D. (2018). Microbiota, a key player in alcoholic liver disease. Clin. Mol. Hepatol. 24, 100–107. doi: 10.3350/cmh.2017.0067 29268595 PMC6038939

[ref3] Hsu C. L. Schnabl B. (2023). The gut-liver axis and gut microbiota in health and liver disease. Nat. Rev. Microbiol. 21, 719–733. doi: 10.1038/s41579-023-00904-3, 37316582 PMC10794111

[ref4] Huang M. Zhang Y. Ni M. Shen M. Tao Y. Shen W. . (2024). Shen-Bai-Jie-Du decoction suppresses the progression of colorectal adenoma to carcinoma through regulating gut microbiota and short-chain fatty acids. Chin. Med. 19:149. doi: 10.1186/s13020-024-01019-4, 39465423 PMC11514841

[ref5] Kanagalingam G. Bajaj J. S. (2024). Gut-microbiome composition and function in progression of alcohol-associated liver disease: going beyond western experiences. Hepatol. Int. 18, 873–875. doi: 10.1007/s12072-024-10677-3 38717692

[ref6] Kang S. Koh J. M. Im D. S. (2024). N-3 polyunsaturated fatty acids protect against alcoholic liver steatosis by activating FFA4 in Kupffer cells. Int. J. Mol. Sci. 25:5476. doi: 10.3390/ijms25105476 38791514 PMC11122576

[ref7] Lesniak N. A. Tomkovich S. Henry A. Taylor A. Colovas J. Bishop L. . (2022). Diluted fecal community transplant restores Clostridioides difficile colonization resistance to antibiotic-perturbed murine communities. MBio 13:e0136422. doi: 10.1128/mbio.01364-22 35913161 PMC9426422

[ref8] Li T. Wu J. Xia S. Yang H. Mu H. Zhu Y. . (2025). Lactiplantibacillus plantarum BD7807 ameliorates high-fat diet-induced lipid metabolic disorders and intestinal dysfunction via SCFAs-GPR43 pathway. Food Res. Int. 220:117108. doi: 10.1016/j.foodres.2025.117108 41074321

[ref9] Liu Y. Li K. Xu J. Shen W. Li Y. Ma J. . (2025). Alpha-linolenic acid ameliorates T2DM via reshaping gut-liver axis and inflammatory GPR120-NF-kappaB/NLRP3 pathway in mouse and rat models. Phytomedicine 147:157214. doi: 10.1016/j.phymed.2025.157214 40929881

[ref10] Liu P. Wang Y. Yang G. Zhang Q. Meng L. Xin Y. . (2021). The role of short-chain fatty acids in intestinal barrier function, inflammation, oxidative stress, and colonic carcinogenesis. Pharmacol. Res. 165:105420. doi: 10.1016/j.phrs.2021.105420 33434620

[ref11] Liu C. Zhou Y. Tu Q. Yao L. Li J. Yang Z. (2023). Alpha-linolenic acid pretreatment alleviates NETs-induced alveolar macrophage pyroptosis by inhibiting pyrin inflammasome activation in a mouse model of sepsis-induced ALI/ARDS. Front. Immunol. 14:1146612. doi: 10.3389/fimmu.2023.1146612 37051243 PMC10083395

[ref12] Philips C. A. Ahamed R. Oommen T. T. Nahaz N. Tharakan A. Rajesh S. . (2024). Clinical outcomes and associated bacterial and fungal microbiota changes after high dose probiotic therapy for severe alcohol-associated hepatitis: an observational study. Medicine (Baltimore) 103:e40429. doi: 10.1097/MD.0000000000040429 39533632 PMC11556976

[ref13] Philips C. A. Pande A. Shasthry S. M. Jamwal K. D. Khillan V. Chandel S. S. . (2017). Healthy donor fecal microbiota transplantation in steroid-ineligible severe alcoholic hepatitis: a pilot study. Clin. Gastroenterol. Hepatol. 15, 600–602. doi: 10.1016/j.cgh.2016.10.029, 27816755

[ref14] Shasthry S. M. (2020). Fecal microbiota transplantation in alcohol related liver diseases. Clin. Mol. Hepatol. 26, 294–301. doi: 10.3350/cmh.2020.0057, 32570299 PMC7364360

[ref15] Szabo G. Bala S. (2010). Alcoholic liver disease and the gut-liver axis. World J. Gastroenterol. 16, 1321–1329. doi: 10.3748/wjg.v16.i11.1321 20238398 PMC2842523

[ref16] Takic M. Ranković S. Girek Z. Pavlović S. Jovanović P. Jovanović V. . (2024). Current insights into the effects of dietary alpha-linolenic acid focusing on alterations of polyunsaturated fatty acid profiles in metabolic syndrome. Int. J. Mol. Sci. 25:4909. doi: 10.3390/ijms25094909 38732139 PMC11084241

[ref17] Tao Z. Wang Y. (2025). The health benefits of dietary short-chain fatty acids in metabolic diseases. Crit. Rev. Food Sci. Nutr. 65, 1579–1592. doi: 10.1080/10408398.2023.2297811 38189336

[ref18] Taur Y. Coyte K. Schluter J. Robilotti E. Figueroa C. Gjonbalaj M. . (2018). Reconstitution of the gut microbiota of antibiotic-treated patients by autologous fecal microbiota transplant. Sci. Transl. Med. 10:eaap9489. doi: 10.1126/scitranslmed.aap9489 30257956 PMC6468978

[ref19] Taylor P. R. Roy S. Leal S. M. Jr. Sun Y. Howell S. J. Cobb B. A. . (2014). Activation of neutrophils by autocrine IL-17A-IL-17RC interactions during fungal infection is regulated by IL-6, IL-23, RORgammat and dectin-2. Nat. Immunol. 15, 143–151. doi: 10.1038/ni.2797, 24362892 PMC3972892

[ref20] Wang J. Zhao Q. Zhang S. Liu J. Fan X. Han B. . (2026). Microbial short chain fatty acids: effective histone deacetylase inhibitors in immune regulation (review). Int. J. Mol. Med. 57, 1–29. doi: 10.3892/ijmm.2025.5687 41201037 PMC12634069

[ref21] Xiang A. Chang Y. Shi L. Zhou X. (2024). Mapping the relationship between alcohol use disorder and gut microbiota: a 20-year bibliometric study. Front. Microbiol. 15:1457969. doi: 10.3389/fmicb.2024.1457969 39624719 PMC11609174

[ref22] Yang C. J. Peng Y. S. Sung P. C. Hsieh S. Y. (2025). Protocol for oral fecal gavage to reshape the gut microbiota in mice. STAR Protoc 6:103585. doi: 10.1016/j.xpro.2024.103585, 39854205 PMC11803845

[ref23] Zhang D. Jian Y. P. Zhang Y. N. Li Y. Gu L. T. Sun H. H. . (2023). Short-chain fatty acids in diseases. Cell Commun. Signal 21:212. doi: 10.1186/s12964-023-01219-9 37596634 PMC10436623

