# Supplementary figures and images for: Fecal microbiota transplantation ameliorates alcohol-associated liver disease through coordinated restoration of short-chain fatty acid and α-linolenic acid signaling
Source: Front Microbiol. 2026 Mar 11;17:1744446. doi: 10.3389/fmicb.2026.1744446 (PMC13013527; doi:10.3389/fmicb.2026.1744446)

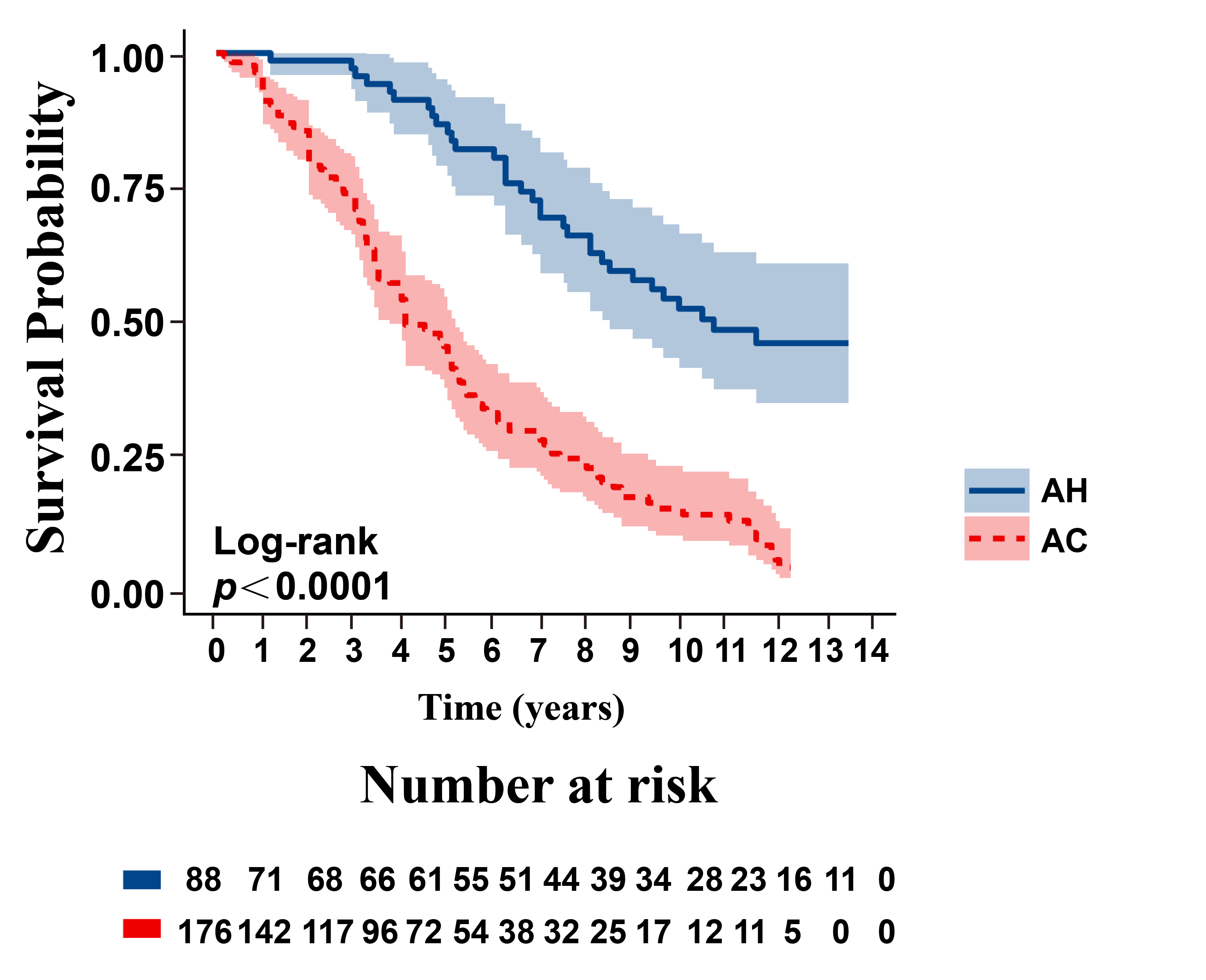

Supplement: SUPPLEMENTARY FIGURE S1 — Survival curves of mice in the AH and AC groups. [file Image_1.JPEG]

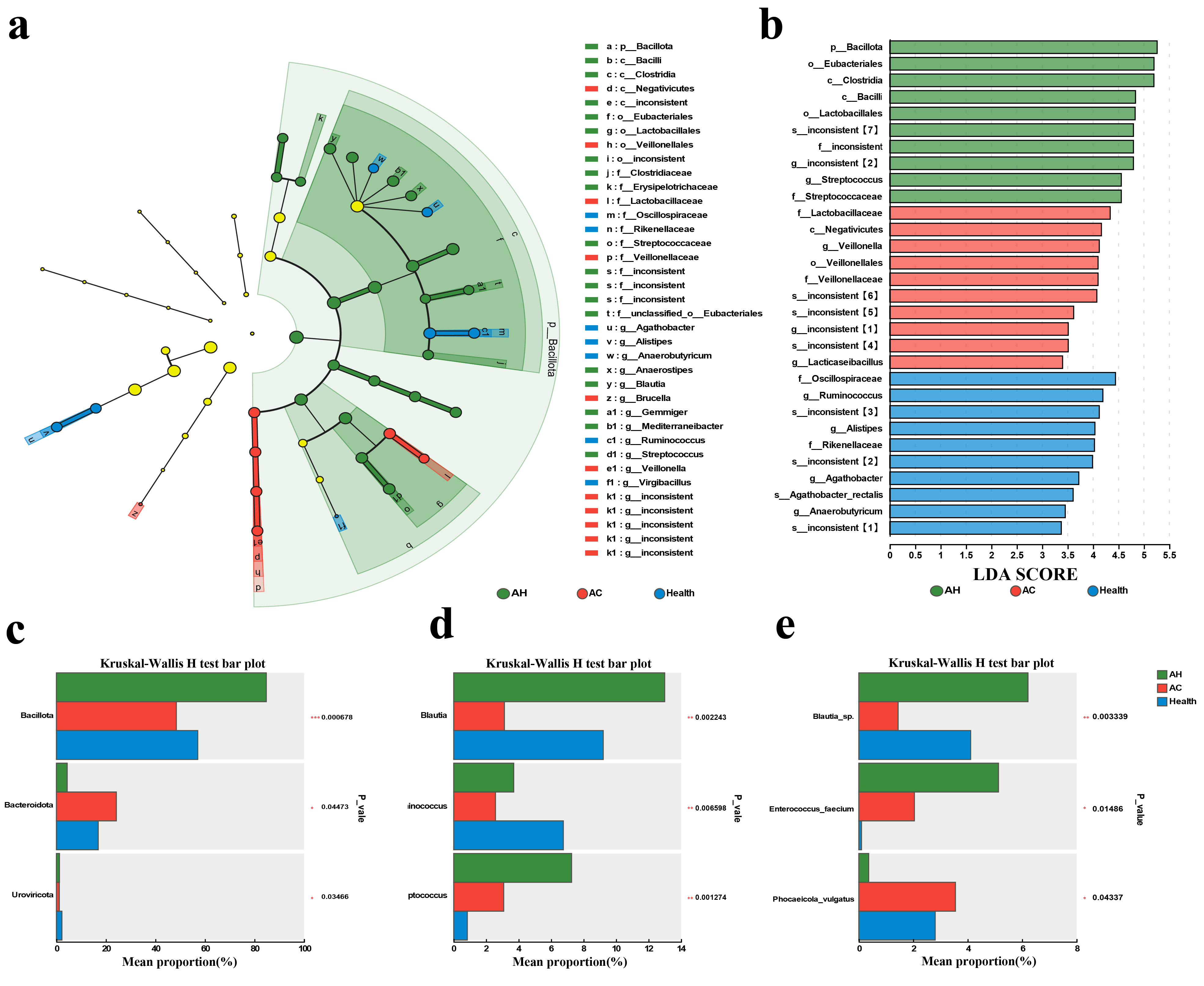

Supplement: SUPPLEMENTARY FIGURE S2 — Differential taxonomic features across different stages of alcohol-related liver disease. (a) LEfSe cladogram analysis: differentially enriched taxa among groups were identified using LEfSe with an LDA score threshold >3.5. The cladogram illustrates the phylogenetic relationships of significantly enriched taxa and their corresponding group associations. (b) LDA scores of discriminative taxa identified by LEfSe: representative taxa that significantly distinguished AH, AC, and healthy control groups are displayed along with their corresponding LDA scores, quantifying their relative contributions to intergroup discrimination. (c–e) Abundance comparisons of representative differential taxa: Selected differentially abundant taxa are presented at the phylum (c), genus (d), and species (e) levels, showing their mean relative abundances across groups. Intergroup differences were assessed using the Kruskal–Wallis test. Bar plots represent mean values, and error bars indicate the standard error of the mean (SEM). [file Image_2.JPEG]

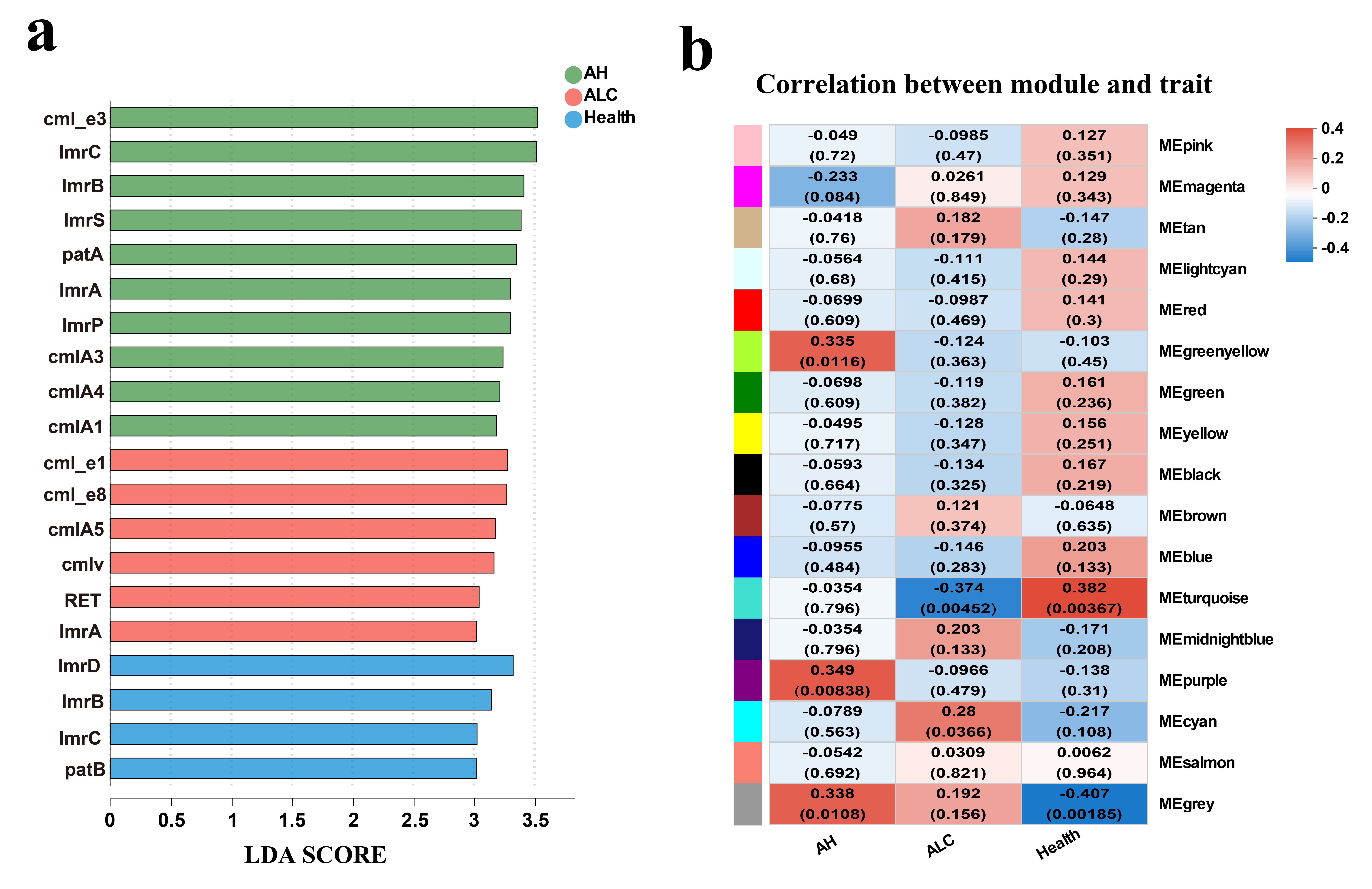

Supplement: SUPPLEMENTARY FIGURE S3 — Functional supportive analysis of the gut microbiota across different stages of ALD. (a) LEfSe analysis based on the Comprehensive Antibiotic Resistance Database (CARD) (LDA >3). Differentially enriched antibiotic resistance genes among groups were identified using LEfSe with an LDA score threshold >3, based on CARD annotations. (b) Weighted Gene Co-expression Network Analysis (WGCNA). WGCNA was performed to identify co-expression modules of microbial genes and to explore their associations with clinical phenotypes across different stages of ALD. [file Image_3.JPEG]

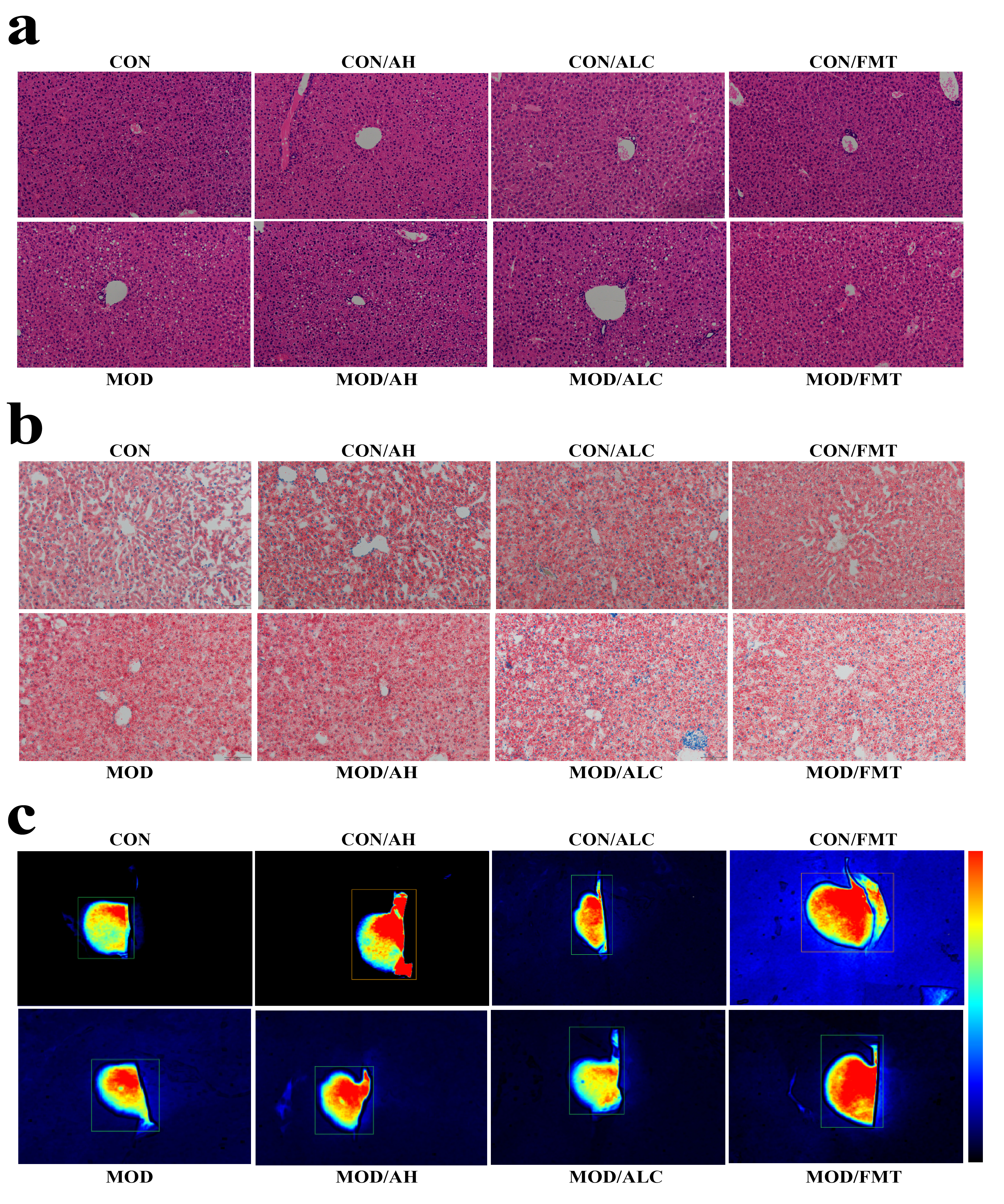

Supplement: SUPPLEMENTARY FIGURE S4 — Effects of FMT from different donor sources on hepatic histological architecture and microcirculatory perfusion in ALD mice. (a) H&E staining. Representative liver sections showing hepatocellular morphological alterations across different groups. (b) Oil Red O staining. Visualization of hepatic lipid accumulation to assess the degree of steatosis. (c) Hepatic perfusion analysis. Laser speckle imaging was used to evaluate the effects of different FMT interventions on hepatic microcirculatory perfusion. Scale bar = 100 μm. [file Image_4.JPEG]

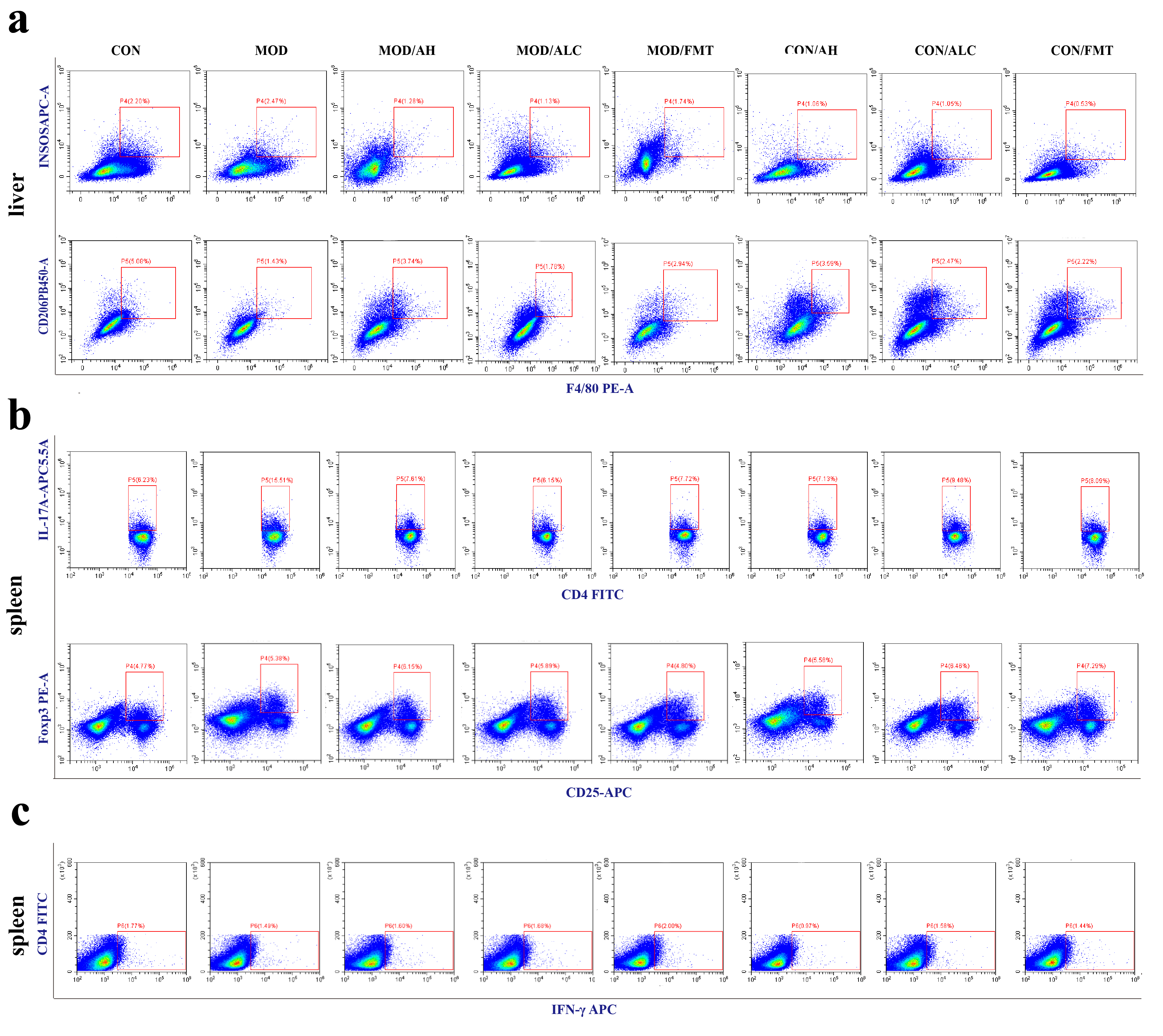

Supplement: SUPPLEMENTARY FIGURE S6 — Flow cytometric analysis of immune cell populations. (a) Hepatic M1 macrophages (F4/80⁺CD11b⁺iNOS⁺) and M2 macrophages (F4/80⁺CD11b⁺CD206⁺). (b) Splenic Th17 cells (CD4⁺IL-17A⁺) and Tregs (CD4⁺CD25⁺Foxp3⁺). (c) IFN-γ–producing T cells (CD4⁺IFN-γ⁺). n ≥ 4 per group. [file Image_6.JPEG]

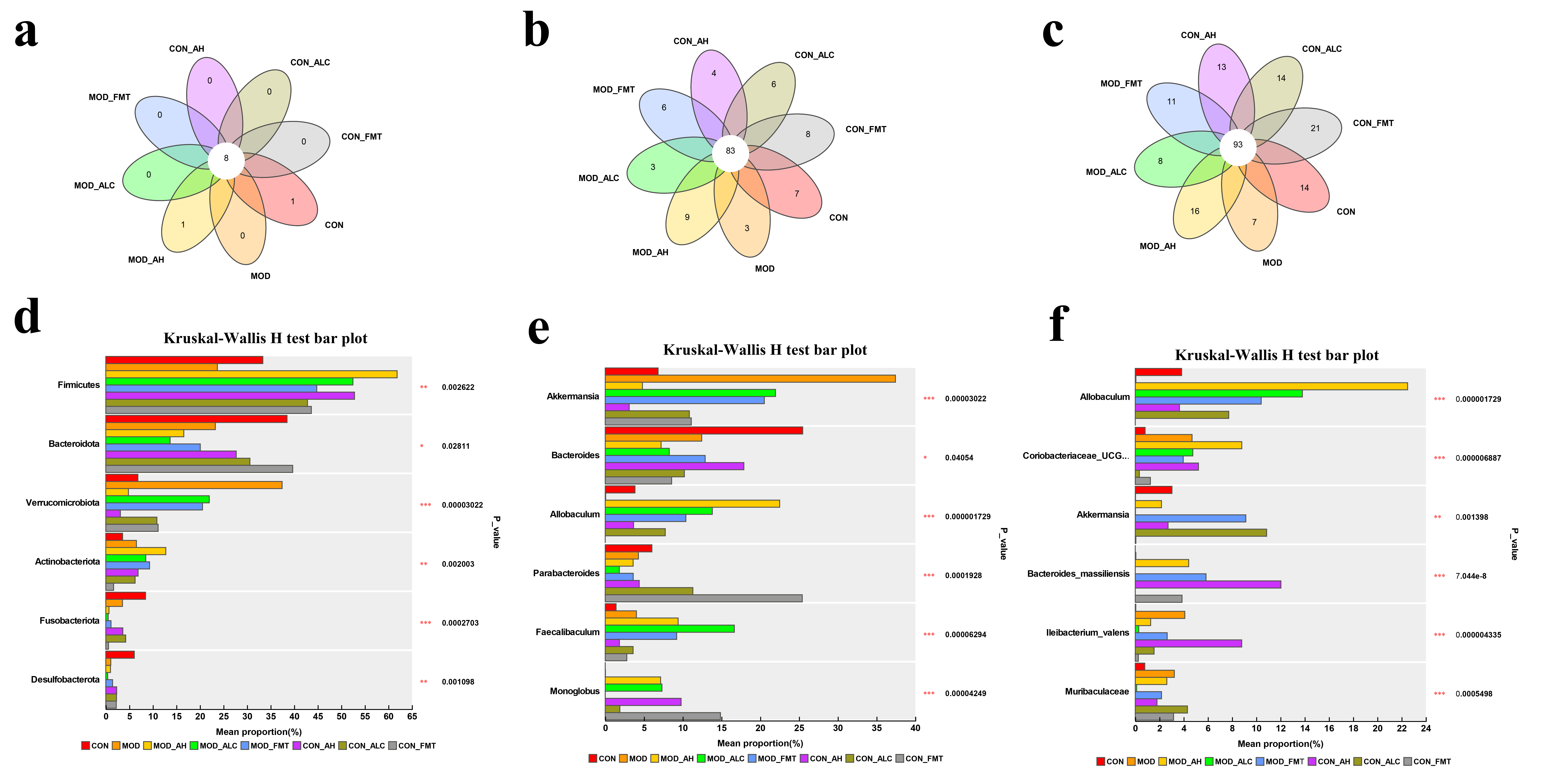

Supplement: SUPPLEMENTARY FIGURE S7 — Taxonomic differences in gut microbiota composition among groups. (a–c) Venn diagrams showing shared and unique ASVs among different groups. (d–f) Relative abundance of gut microbial taxa at the phylum, genus, and species levels. n ≥ 5 per group. Data are presented as mean ± SD. Statistical comparisons among multiple groups were conducted using one-way ANOVA or Kruskal–Wallis test as appropriate, followed by post hoc multiple-comparison analysis. * P < 0.05, ** P < 0.01, *** P < 0.001. [file Image_7.JPEG]
