# Supplementary material for: Fecal microbiota transplantation ameliorates alcohol-associated liver disease through coordinated restoration of short-chain fatty acid and α-linolenic acid signaling
Source: Front Microbiol. 2026 Mar 11;17:1744446. doi: 10.3389/fmicb.2026.1744446 (PMC13013527; doi:10.3389/fmicb.2026.1744446)
Supplement: Supplementary file 10 [file Table_1.docx]

## ****Table S1.** Donor metadata and microbiome characteristics**

| Variable | AH (n = 8) | AC (n = 23) | Healthy (n = 39) |
| --- | --- | --- | --- |
| Age (years) | 46.8 ± 7.4 | 52.3 ± 8.9 | 48.6 ± 6.2 |
| Male, n (%) | 6 (75%) | 17 (74%) | 29 (74%) |
| BMI (kg/m²) | 25.1 ± 2.3 | 24.6 ± 2.8 | 23.9 ± 2.1 |
| Current alcohol intake (g/day) | 122 ± 38 | 96 ± 27 | 5 ± 3 |
| Duration of heavy drinking (years) | 12.4 ± 4.2 | 18.7 ± 6.5 | – |
| Recent antibiotic use (3 months), n (%) | 1 (12.5%) | 4 (17%) | 0 |
| Proton pump inhibitor use, n (%) | 2 (25%) | 9 (39%) | 0 |
| β-blocker use, n (%) | 0 | 11 (48%) | 0 |
| Lactulose use, n (%) | 1 (12.5%) | 8 (35%) | 0 |
| Serum ALT (U/L) | 168 ± 52 | 74 ± 29 | 24 ± 8 |
| Total bilirubin (mg/dL) | 6.2 ± 2.1 | 3.4 ± 1.8 | 0.8 ± 0.2 |
